# Focused ultrasound-augmented targeting delivery of nanosonosensitizers from homogenous exosomes for enhanced sonodynamic cancer therapy

**DOI:** 10.7150/thno.33183

**Published:** 2019-07-09

**Authors:** Yichen Liu, Lianmei Bai, Kaili Guo, Yali Jia, Kun Zhang, Quanhong Liu, Pan Wang, Xiaobing Wang

**Affiliations:** Key Laboratory of Medicinal Resources and Natural Pharmaceutical Chemistry, Ministry of Education, National Engineering Laboratory for Resource Developing of Endangered Chinese Crude Drugs in Northwest of China, College of Life Sciences, Shaanxi Normal University, Xi'an, Shaanxi, China.

**Keywords:** Nanosonosensitizer, Sinoporphyrin sodium, Exosome, Ultrasound-responsive drug release, Sonodynamic therapy

## Abstract

Sonodynamic therapy (SDT), wherein focused ultrasound is used to guide the site-specific delivery of nano-sonosensitizers and trigger profound sono-damage, has great potential in cancer theranostics. The development of nanosensitizers with high sono-activatable efficiency and good biosafety is however challenging.

**Methods**: In this study, we designed a functionalized smart nanosonosensitizer (EXO-DVDMS) by loading sinoporphyrin sodium (DVDMS), an excellent porphyrin sensitizer with both potential therapeutic and imaging applications, onto homotypic tumor cell-derived exosomes. Because of the high binding-affinity between DVDMS and proteins, coincubation of DVDMS and exosome would result in DVDMS attached on the surface or loaded in the core of exosomes. The prepared EXO-DVDMS was applied for ultrasound-responsive controlled release and enhanced SDT.

**Results**: Tumor cell-derived exosomes exhibited high stability and specificity towards the homotypic tumors, along with highly controlled ultrasound-responsive drug release, and boosted reactive oxygen species (ROS) generation to augment SDT. Intriguingly, EXO-DVDMS was endocytosed by lysosomes, and the low pH in the latter triggered DVDMS relocation synergistically with the ultrasound, thereby initiating multiple cell death-signaling pathways. Furthermore, the exosomal formulation served as a functionalized nanostructure, and facilitated simultaneous imaging and tumor metastasis inhibition, that were respectively 3-folds and 10-folds higher than that of free form.

**Conclusions**: Taken together, our findings suggest that an extracorporeal ultrasound device can non-invasively enhance homogenous tumor targeting and SDT toxicity of EXO-DVDMS, and the developed endogenous nano-sonosensitizer is a promising nanoplatform for activated cancer theranostics.

## Introduction

Cancer is a serious public health concern worldwide, and considerable efforts have been made over the years to develop efficient and safe therapeutic modalities 1,2. Photodynamic therapy (PDT) is a minimally invasive treatment against aggressive tumors, but shows limited anti-tumor efficacy due to low *in vivo* penetration 3. Sonodynamic therapy (SDT) is a novel approach based on ultrasound (US), and has recently gained widespread interest due to its easy-implementation, and the intrinsically high tissue-penetration of US 4-6. The possible underlying mechanisms of SDT action include ultrasonic cavitation, production of free radicals, apoptosis, or a combination of any of these 7,8.

Sonosensitizers are critical factors in SDT, and are classified into the organic and inorganic types 4. Inorganic sonosensitizers, such as titanium oxide (TiO_2_) nanoparticles (NPs) 9, 10, hollow mesoporous silica (HMSNs) NPs and metal-organic framework (MOF) NPs 7,11-16, have unique energy-band structures, large surface area for drug/gas loading and relatively high chemical/physiological stability, but have biodegradability and biosafety issues that limit their clinical translation 17, 18. Organic sonosensitizers include natural porphyrins and their derivatives like photofrin 8, DCPH-P-Na 19, hematoporphyrin monomethyl ether, etc. 20, which show satisfactory biodegradation, high biocompatibility and easy fabrication. They have been studied as potential anti-cancer therapeutics for several decades, and some have entered clinical testing. However, conventional organic sonosensitizers usually suffer from low water solubility, low chemical and biological stability, poor tumor accumulation and rapid clearance from the bloodstream, resulting in less than optimal concentration at the tumor sites and lower therapeutic efficacy 7, 21. Recent advances in the combined use of nanomedicine and PDT, which include nanoparticle conjugates like SPIO NPs, semiconductor quantum dots and gold NPS, show great promise in overcoming the disadvantages of bare porphyrins and achieving an integrated platform for diagnosis and enhanced therapeutic outcome 22. In contrast, there are only few reports available on ultrasound triggered nano-porphyrins, and their clinical translation is stymied due to the underlying immunogenicity, cytotoxicity of supporter molecules and the degradation products 23, 24.

To overcome the limitations of conventional delivery systems, increasing efforts have been focused on developing carriers derived from endogenous cells or subcellular structures. Exosomes are nanoscale (30-150 nm) cell-derived membrane vesicles that carry macromolecules from their parental cells, and mediate cell-to-cell communication 25, 26. Exosomes have been developed for use as diagnostic markers in various malignancies, and also as delivery vehicles for anti-cancer chemotherapeutics and nucleic acids. Their endogenous origin imparts excellent biocompatibility and the ability to evade uptake by the reticuloendothelial system (RES), which protect the encapsulated drug till delivery to the lesion site 27-31. Furthermore, several clinical trials using exosomes for immunotherapy have already demonstrated the safety of exosome administration in humans 32, 33. However, to the best of our knowledge, exosome based nano-sonosensitizers have not been developed so far. Additionally, in spite of the potential biocompatibility and inherent targeting ability, the application of exosomes still faces severe challenges. The targeting efficiency of exosomes partly relies on the enhanced permeability and retention (EPR) effect that is highly heterogeneous among different tumors, leading to insufficient drug accumulation in tumors. Besides, compared with synthetic materials, the function of exosomes is less versatile, for example, it cannot respond to exogenous stimulations, which limits their application in drug controlled release 34, 35. Therefore, improved exosome-based nanoparticles with stimuli-responsive ability, enhanced accumulation/penetration, and endogenic biological advantages, would be of great importance in precision drug delivery and thus can be adapted for targeted SDT strategy.

**Scheme 1:**
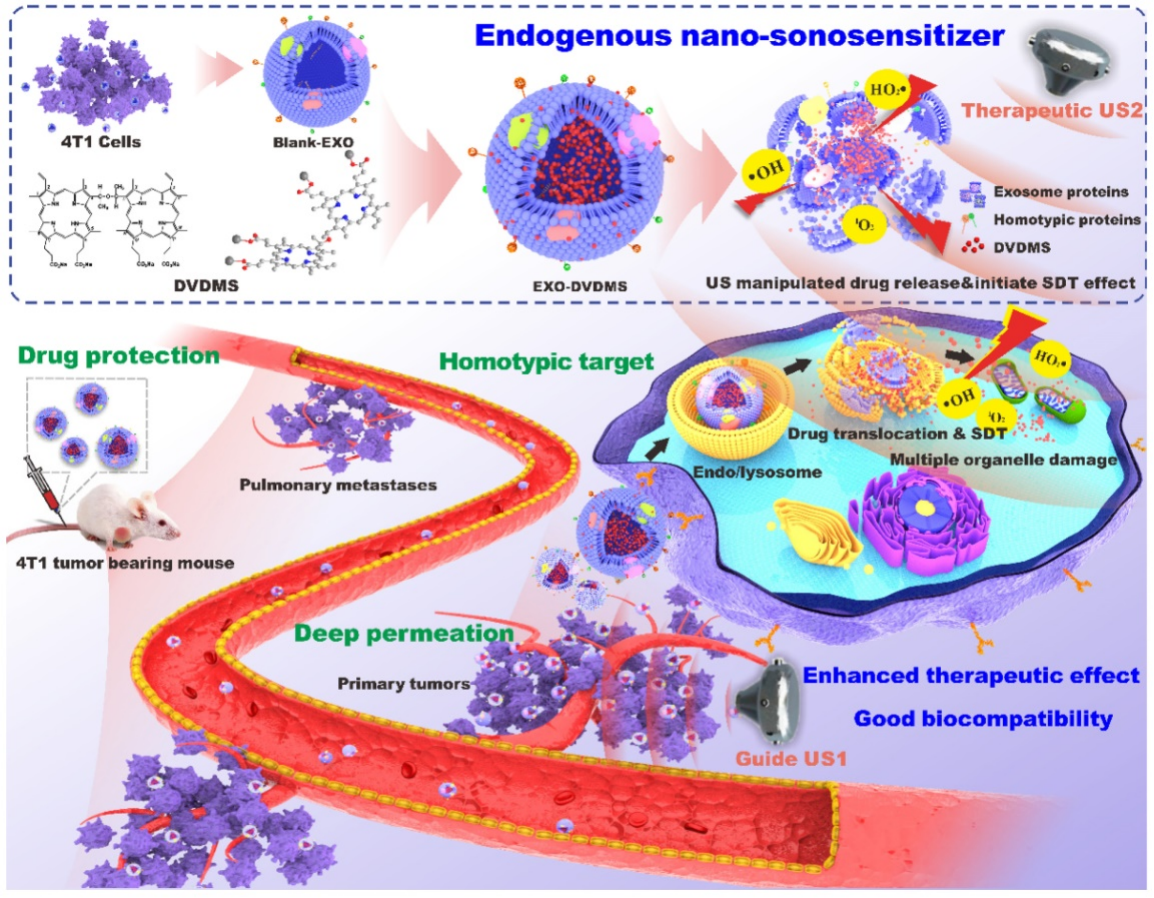
** Illustration of endogenous nanosonosensitizers for focused US-augmented targeting delivery to recognize homotypic cancer cell, stimuli-responsive drug release, and enhanced SDT.** EXO-DVDMS were prepared by uploading DVDMS onto 4T1 cell derived exosomes via a very mild and green method without causing any surface changes. Through guide US1 and therapeutic US2, EXO-DVDMS exhibited specific accumulation in both the primary tumor and metastatic lesions, and achieved ultrasound manipulated drug relocation and effective SDT after intravenous injection.

Herein, we designed a functionalized smart nanoparticle system by loading sinoporphyrin sodium (DVDMS), an excellent porphyrin sonosensitizer with potential therapeutic and imaging applications 36-39, onto homotypic tumor cell-derived exosomes **(Scheme 1)**. Being well camouflaged of tumor exosomes, the resulting EXO-DVDMS was harnessed as fluorescence-guided “spy drone” for improved drug stability in bloodstream/tumor microenvironment and enhanced targeting delivery of DVDMS to the primary as well as metastatic tumors. A guided-ultrasound (US1) was first introduced to promote local accumulation of EXO-DVDMS in tumor region, subsequently, the therapeutic-ultrasound (US2) was applied for SDT. EXO-DVDMS exhibited controlled ultrasound-responsive drug release and enhanced ROS generation which augmented SDT both *in vivo* and *in vitro*. In addition, the intracellular transportation patterns of EXO-DVDMS under ultrasonic stimulation and conditions imitating the tumor microenvironment were also investigated. The ultrasound-manipulated EXO-DVDMS platform provides insights into the application of cell-derived vesicles as spatio-temporally controlled nanosensitizers and drug delivery strategies, in order to achieve safe, accurate, effective and personalized anti-cancer therapy.

## Results and Discussion

### Preparation and characterization of EXO-DVDMS

**Figure 1:**
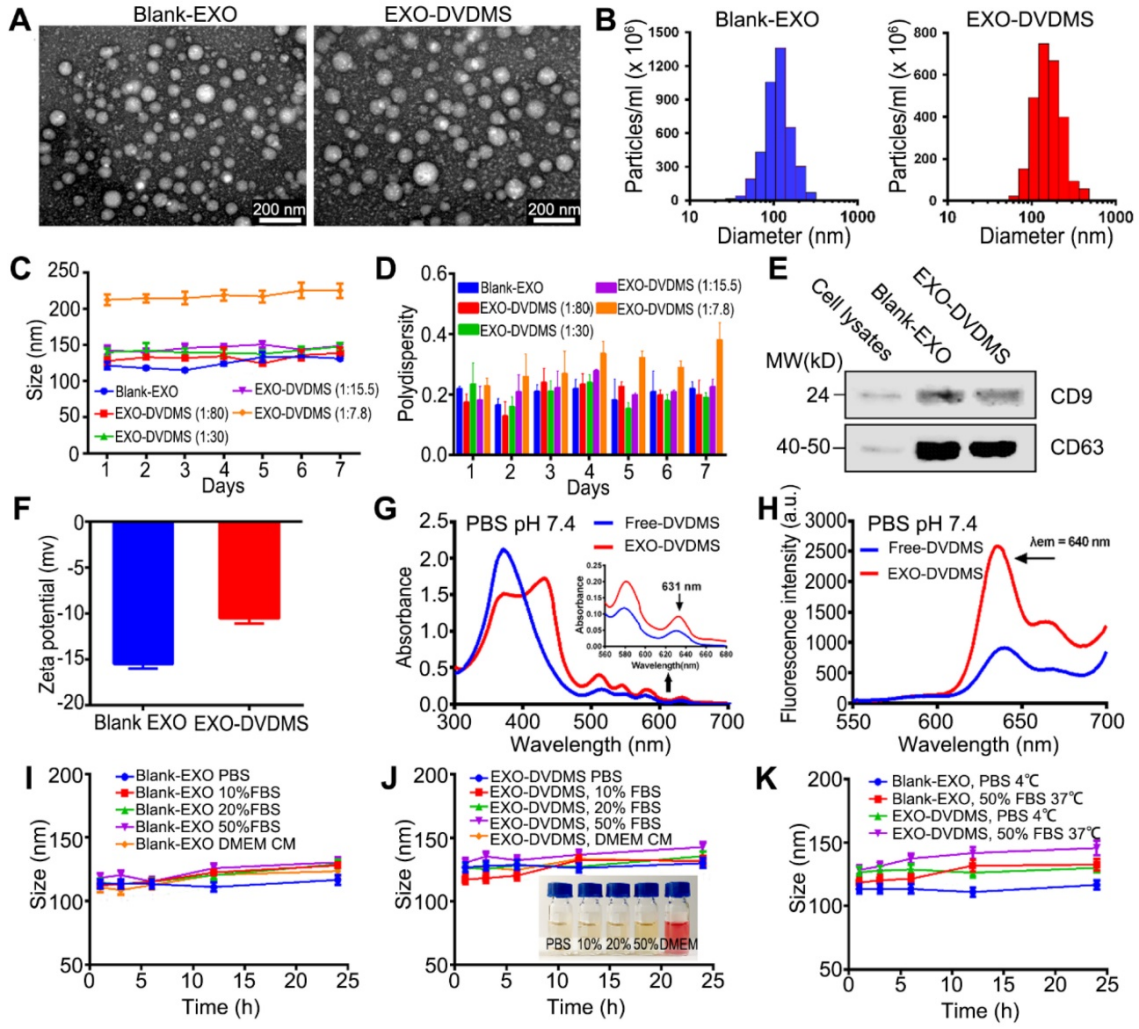
** Identification and characteristics of EXO-DVDMS. (A)** TEM images of Blank-EXO and EXO-DVDMS. Scale bar, 200 nm. **(B)** Size distributions histogram of Blank-EXO and EXO-DVDMS based on NTA measurement. **(C)** Changes of size distributions and **(D)** PDI of EXO-DVDMS with different mass ratio (DVDMS: exosome = 1:7.8, 15.5, 30, 80) in a week based on DLS analysis. **(E)** Western blot analysis of CD9 and CD63 from 4T1 cells, Blank-EXO and EXO-DVDMS. **(F)** Zeta potential of Blank-EXO and EXO-DVDMS. **(G)** Absorption spectra and fluorescence spectra **(H)** of Free-DVDMS (blue), EXO-DVDMS (red) at DVDMS concentration of 20 µg/mL. Changes of size distribution of Blank-EXO **(I)** and EXO-DVDMS **(J)** after different storage times in the presence of serum. Inset: photographs of EXO-DVDMS after incubation in different medium for 24 h. **(K)** Changes of size distribution of Blank-EXO and EXO-DVDMS after different storage times at 4 °C or 37 °C based on DLS analysis. Error bars represent the S.D. for n = 3.

Exosomes were isolated from the supernatant of 4T1 cells by specially-made exosomes isolation reagent based on previous studies with some modifications 40, 41. DVDMS as the US responsive agent was loaded into 4T1 cell derived exosomes via a very mild and green method. The uploaded DVDMS with different distribution (encapsulated in core / inserted in membrane) was designed for sequenced functions including responsive drug release and SDT when different US level was applied **(Scheme 1)**. Based on the protein concentration, 4-8 µg of exosomes were harvested per 10^6^ mammary cancer 4T1 cells. Transmission electron microscopy (TEM) imaging showed that the unmodified exosomes (blank-EXO) had diameters ranging from 40-150 nm **(Figure 1A)**, and nanoparticle tracking analysis (NTA) showed a hydrodynamic diameter distribution with an average dimension of 112.52 ± 4.68 nm **(Figure 1B, Figure S1)**, consistent with previous reports 25. Tetraspanin CD9 and CD63, as standard markers for extracellular vesicles 42, 43, were abundant in the obtained exosomes (**Figure 1E**). Potential structural damage in single-pass freeze-thawed exosomes were also analyzed, and as shown in **Figure S2**, the majority did not have any morphological impairment, with average diameter 118.32 ± 5.68 nm.

DVDMS was loaded efficiently onto the exosomes without causing any surface modifications in the latter, after testing and optimizing the different parameters including mass ratio (**Table S1**), incubation time (**Table S2**) and temperature (**Table S3**). The encapsulation efficiency (EE%) and drug loading efficiency (DL%) of EXO-DVDMS prepared by different parameters were calculated by a standard curve of DVDMS concentration to the fluorescence intensity (λex = 410 nm, λem = 627 nm) **(Figure S3)**. As shown in **Figure 1C-D** and **Table S1**, blank-EXO and EXO-DVDMS of different mass ratios retained their small size and polydispersity index (PDI) in PBS at 4°C for at least 7 days **(Figure S4)**. The optimal EXO-DVDMS was prepared by co-incubation of DVDMS and 4T1 derived exosomes at a mass ratio of 1:15.5 for 30 min at 25℃with relatively high drug loading capacity (EE% = 84.75%, DL% = 5.18%, 5.46 µg DVDMS loading per 100 µg exosomal protein), low polydispersity (0.18 ± 0.05), and suitable size distribution (mean size at 126.71 ± 3.86)(**Table S1-3**).

TEM and NTA (**Figure 1A-B; Figure S1, right**) demonstrated a homogenous morphology and ~10 nm larger average hydrodynamic diameter (126.71 ± 3.86 nm) of EXO-DVDMS compared to blank-EXO. DVDMS loading also mildly increased the zeta potential of exosomes from -15.48 ± 0.48 mv to -10.67 ± 0.52 mv **(Figure 1F)**, which may be due to the integration of the sonosensitizer into the vesicle membranes, but did not affect the expression levels of CD9 and CD63 **(Figure 1E)**.

The absorption spectra of Free-DVDMS and EXO-DVDMS are shown in **Figure 1G**. Free-DVDMS in PBS (20 µg/mL) exhibited a strong Soret peak at ~370 nm, and Q-bands between 500 and 700 nm. The UV-vis spectrum of EXO-DVDMS shows a superimposition of DVDMS peaks and an enhanced absorbance on Q-bands, especially at 631 nm, indicating successful DVDMS loading. However, the characteristic peaks of the loaded DVDMS did not completely match that of pure DVDMS, and showed a new peak at 430 nm accompanied by the decrease of Soret peak. In our previous studies, we observed a strong spontaneous interaction between DVDMS and proteins (e.g. BSA) on at least one independent binding site in aqueous solution 44. Therefore, we hypothesize that the new peak could be due to the two sterically hindered porphyrin rings of DVDMS interacting with the exosomal proteins, which results in one surface-attached and one free porphyrin ring 37, 38. The fluorescence spectra of free-DVDMS and EXO-DVDMS are shown in **Figure 1H**. DVDMS showed fluorescence emission peak at ~640 nm, which did not change upon loading onto exosome, indicating that the fluorescence spectral properties of DVDMS are not significantly affected by the carrier. Interestingly, however, the fluorescence intensity of the loaded DVDMS was significantly enhanced via intramolecular charge transfer, which is highly promising for real-time visualization of *in vivo* DVDMS delivery and distribution, and molecular imaging-guided SDT.

Both blank-EXO and EXO-DVDMS exhibited excellent colloidal stability, with no visible precipitation in either complete DMEM or PBS containing 10%, 20% or 50% serum at 4°C or 37°C even after 24 h **(Figure 1I-K)**, indicating good biocompatibility of the particles. Taken together, no major damages were seen in the DVDMS loaded exosomes in terms of size, morphology, immunophenotype and biostability, suggesting that the DVDMS loading protocol was successful. The good stability combined with small size are highly conducive to the circulation and targeted accumulation of EXO-DVDMS.

### Exosomal encapsulation improved DVDMS stability and enhanced singlet oxygen yield

The interactions between porphyrins and the endogenous biomacromolecules are unavoidable, and can affect the physical and chemical properties of sonosensitizers and thus decrease the SDT efficacy 44. For example, recent spectroscopic studies show poor stability of DVDMS at acidic pH 45. This is highly relevant in the context of drug delivery since interactions with various serum proteins as well as the acidic condition of tumor micro-environment and lysosomes can result in inactivation and/or degradation of the drugs. One of the advantages of using an exosomal carrier is that it protects the uploaded cargo against an unfavorable environment till it is delivered to the target site.

**Figure 2:**
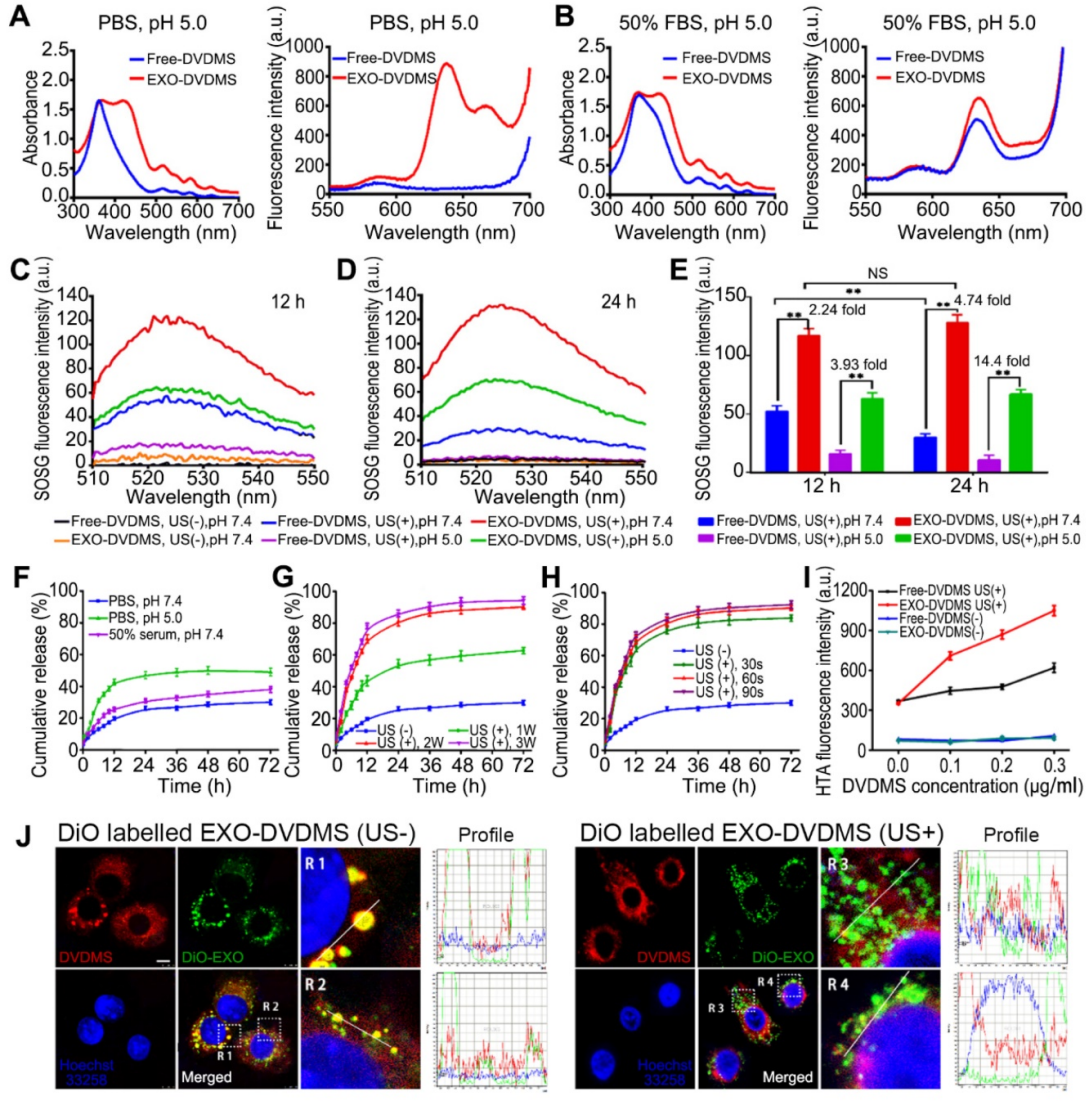
** Spectral analysis, single oxygen production and DVDMS release from EXO-DVDMS initiated by US and low pH.** Absorption spectra (left) and fluorescence spectra (right) of DVDMS and EXO-DVDMS in **(A)** PBS buffer pH=5.0, or** (B)** with the presence of 50% FBS, pH=5.0. **(C)** Fluorescence emission spectra of SOSG (5.0 µΜ) solution mixed with Free-DVDMS and EXO-DVDMS at pH values of 7.4 and 5.0 after 12 h or **(D)** 24 h incubation with US exposure (3 W, 1.0 MHz, 60 s). **(E)** Data statistics of fluorescence emission spectra of SOSG in (C) and (D).** (F)** Drug release of EXO-DVDMS under 50% serum and different pH value. **(G)** Drug release of EXO-DVDMS after exposure to different acoustic intensities (LP=1, 2 and 3 W) for 60 s under pH value of 7.4. **(H)** Drug release of EXO-DVDMS after exposure to different acoustic duration (30, 60 and 90 s) at 2 W. **(I)** US cavitation effect with the increase of drug dose (Free-DVDMS or EXO-DVDMS) by TA method (3 W, 60 s). **(J)** Intracellular dissociation of DVDMS from EXO-DVDMS under US exposure (3 W, 60 s), Scale bar=10 µm. ** p<0.01 between groups. Error bars represent the S.D. for n = 3.

As shown in **Figure 2A-B**, the pH and serum concentration of the buffer significantly affected the spectra of DVDMS but not that of EXO-DVDMS, consistent with previous findings on free-DVDMS 44, 45. The fluorescence emission spectrum of free-DVDMS in the presence of serum was a result of the random and unstable interactions between DVDMS and the serum proteins that stabilize the physical properties of the former. Interestingly, EXO-DVDMS maintained its strong Soret absorption peak and characteristic fluorescence emission spectrum even at low pH and 50% fetal bovine serum (FBS), indicating the protective effect of exosomes. In addition, the exosomal cover also potentially minimized the random interactions between DVDMS and serum proteins in circulation, which prevented *in vivo* aggregation.

SDT depends on the synergistic effects of ultrasound and the sonosensitizer. The free radicals derived from the sonosensitizers following US stimulation, such as singlet oxygen (^1^O_2_), superoxide anion (O_2_^-^), hydrogen peroxide (H_2_O_2_) and hydroxyl radical (**^.^**OH), are highly cytotoxic and contribute to the anti-cancer outcome of SDT 46, 47. The levels of reactive oxygen species (ROS) generated from the EXO-DVDMS and Free-DVDMS in neutral and low pH conditions were measured in terms of the fluorescence intensity of singlet oxygen sensor green (SOSG) at 525 nm. At neutral pH (pH = 7.4), EXO-DVDMS showed 2.24- and 4.74-times higher singlet oxygen yield compared to Free-DVDMS at 12 h and 24 h respectively, suggesting more physiological stability of EXO-DVDMS. Remarkably, the singlet oxygen yield of EXO-DVDMS at low pH (pH = 5) were 3.93 and 14.4 times higher than that of Free-DVDMS at 12 h and 24 h **(Figure 2C-E)**, indicating the exosomal coating improved the stability of free DVDMS in tumor microenvironment, and EXO-DVDMS could perform better SDT efficiency than the naked DVDMS. The decreased photometric values of Free-DVDMS under acidic pH (**Figure S5**) should account for the reduced ^1^O_2_ yield. Whereas, the absorbance and emission values of EXO-DVDMS were less influenced by the variable pH. Meanwhile, EXO-DVDMS displayed much higher storage stability than Free-DVDMS, in response to temperature changes (4, 25 and 37℃) (**Figure S6**). Therefore, our newly developed endogenous nanosensitizers are highly stable in stored or physiological conditions, and are beneficial to the following SDT.

### Ultrasound and low pH boosted DVDMS release from EXO-DVDMS

We determined the drug-release profiles of EXO-DVDMS in simulating the physiological environment (pH 7.4), tumor microenvironment (pH 6.5), and late endosome/lysosome conditions (pH 5.0). As shown in **Figure 2F and Figure S7,** EXO-DVDMS released 28.75%, 31.12% and 35.04% of the DVDMS respectively in serum-free PBS (pH 7.4), serum-free PBS (pH 6.5) and in the presence of 50% FBS (pH 7.4) after 48 h, indicating a fairish stability of EXO-DVDMS. This may be due to the outer shell of exosomes could decrease DVDMS' degradation or inactivation in *in-vivo* circulation. At low pH, around 50.23% of the DVDMS were released after 48 h, suggesting DVDMS could be partially dissociated from the exosomal formulation after entering the acidic environment of late endosomes/lysosomes of cancer cells.

The EXO-DVDMS was not only a nano-sonosensitizer, but also an ultrasound-responsive drug delivery platform. As shown in **Figure 2G-H**, the rate of drug release was only 19.79 % within the first 12 h in the absence of any stimulation, while over 43.69%, 70.22% and 78.23% of DVDMS was released after 12 h following 60 s of ultrasonic stimulation of varying intensities **(Figure 2G)**. The sonication time did not significantly affect drug release, which was approximately 65.69%, 70.22%, and 73.33% after 30, 60 and 90 s of stimulation respectively at 12 h after 2 W (load power, LP) **(Figure 2H)**. During the next 60 h, DVDMS in formulations maintained sustained release in different groups until the end of the test. The possible mechanisms underlying DVDMS release upon ultrasonic stimulation including lipid peroxidation, shear force and the cavitation effect 5. US cavitation is known to trigger free radicals generation, which can potentially affect the stability of lipid membranes of the carrier 39. Thus, we measured **^.^**OH level using the TA method, which indirectly demonstrates cavitation event. As shown in **Figure 2I**, the ultrasonic parameters used in this study caused cavitation in both free and encapsulated DVDMS. The ·OH content in the EXO-DVDMS suspension increased in a DVDMS-dose dependent manner after US stimulation, and showed significantly higher HTA fluorescence compared with that of the free-DVDMS, indicating that exosomal DVDMS plus US can increase cavitation, followed by the enhanced drug release and SDT. Unfortunately, not much is known regarding such nanoDVDMS changes in an acoustic field. In this study, we primarily evaluated the morphological changes of Blank-EXO and EXO-DVDMS using TEM observation, which showed that Blank-EXO had no visible changes before and after US, while obviously structural damage was shown in EXO-DVDMS (US+) group, suggesting EXO-DVDMS has good sonosensitivity (**Figure S8**).

To further investigate the ultrasonic spatio-temporal manipulation of EXO-DVDMS, we tracked the intracellular distributions of exosomes and DVDMS. As shown in **Figure 2J**, strong exosomal (green) and DVDMS (red) signals were scattered within the cytoplasm in both the stimulated and unstimulated cells. The unstimulated cells showed extensive co-localization (yellow signals) of the carriers and DVDMS, indicating a slow drug-release pattern without US trigger. However, the co-localized signals largely disappeared after US stimulation, demonstrating an ultrasound-responsive dissociation of DVDMS from the exosomes.

### Cell uptake and targeted delivery of EXO-DVDMS

**Figure 3:**
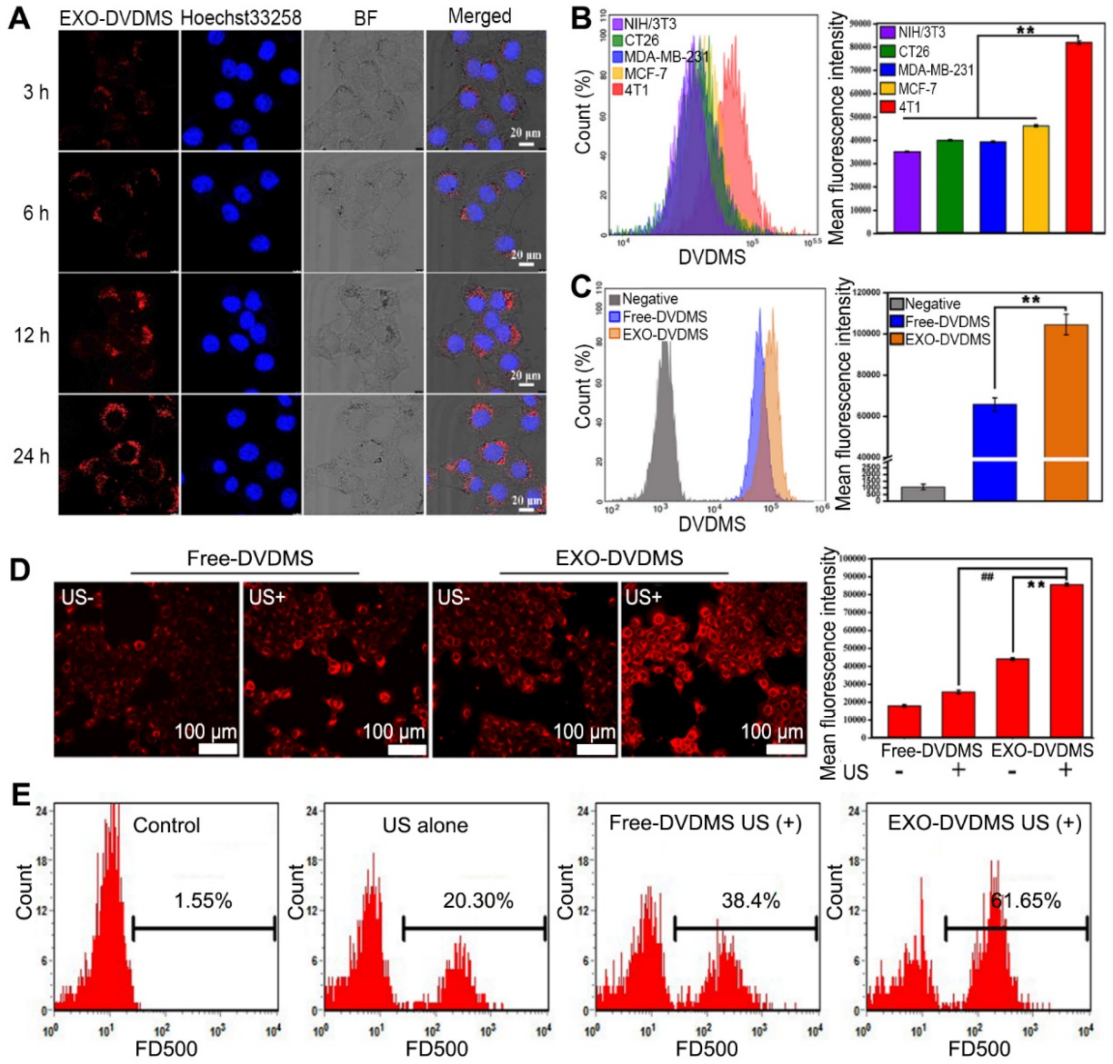
** Cell uptake of EXO-DVDMS. (A)** Intracellular fluorescence of DVDMS was observed using confocal microscopy at the indicated time points after incubation with EXO-DVDMS (DVDMS concentration: 5 µg/mL). **(B)** Cellular uptake of five distinct cell lines (NIH/3T3, CT26, MDA-MB-231, MCF-7 and 4T1 cells) upon 12 h incubation with EXO-DVDMS (left: Flow cytometric profiles, right: Mean fluorescence intensity of different cells). **(C)** Cellular uptake of Free-DVDMS and EXO-DVDMS upon 12 h incubation (left: Flow cytometric profiles, right: Mean fluorescence intensity). **(D)** *In vitro* cellular uptake of Free-DVDMS and EXO-DVDMS with or without US exposure (2 W, 60 s) using CLSM, scale bar = 100 µm (left) and quantification of intracellular uptake (right). **(E)** Detection of cell membrane permeability by FD500-uptake assay. 4T1 cells incubated with Free-DVDMS and EXO-DVDMS were sonicated (2 W, 60 s) in the presence of 1 mg/mL of FD500 conjugated with fluorescein FITC and then the FD500-positive cells were quantified by flow cytometry. ** p<0.01 between groups. Error bars represent the S.D. for n = 3.

The uptake pattern of 4T1-derived exosomes by the homologous cells was investigated using DiO-labeled exosomes. As shown in **Figure S9**, 150 µg/mL of the exosomes showed higher cellular uptake after 3, 6, 12 and 24 h of incubation compared to 75 µg/mL, with respective cellular uptake efficiencies of 21%, 54.27%, 86.67% and 97.83%, indicating that the exosomes were internalized in a time- and concentration-dependent manner. The cellular internalization of EXO-DVDMS was also visualized by the red fluorescence of DVDMS. In line with the cellular uptake pattern of DiO labeled exosomes (**Figure S9A**), EXO-DVDMS was dispersed in the cytoplasm after 3 h, and gradually increased after 6, 12 and 24 h **(Figure 3A)**. To verify the intrinsic specificity of EXO-DVDMS for its homotypic cells, it was tested on the mouse embryo fibroblast NIH/3T3 cells, human breast cancer MCF-7 cells, human breast cancer MDA-MB-231 cells, mouse colon carcinoma CT26 cells and mouse mammary cancer 4T1 cells. Each cell type was incubated with 1 µg/mL EXO-DVDMS for 12 h. As expected, 4T1 cells exhibited the highest uptake efficiency, with 2.35, 2.05, 2.08 and 1.81-fold higher fluorescence intensities compared to that of the NIH/3T3, CT26, MDA-MB-231 and MCF-7 cells, respectively **(Figure 3B)**. Thus, the EXO-DVDMS possess intrinsically superior selectivity to its homologous tumor cells *in vitro*.

The cellular delivery efficiency of Free-DVDMS and EXO-DVDMS were also compared. The fluorescence intensity of the latter was significantly higher (1.62 fold) than the former (p<0.05) **(Figure 3C)**, suggesting the exosomal formulation increased the DVDMS uptake in homotypic cells. Furthermore, US stimulation increased the cellular uptake of DVDMS, with significantly higher uptake of the exosomal-encapsulated compared to the free-DVDMS (p<0.01) **(Figure 3D)**. Although the internalization of drug-loaded exosomes into target cells via endocytosis and membrane fusion has been extensively studied, the potential mechanism of EXO-DVDMS uptake under US stimulation is more complex 5. In addition to endocytosis, mechanical and cavitational effects can facilitate pore formation in the plasma membrane, and possibly mediate the enhanced delivery 48. Likewise, the localized high-speed micro-jet produced by cavitation may also increase the diffusion of drug molecules. As shown in **Figure 3E**, the proportion of 4T1 cells with high FD500 fluorescence intensity increased by 20.3% after US stimulation, with higher frequencies seen in cells incubated with EXO-DVDMS (61.65%) compared to Free-DVDMS (38.40%; p<0.01), indicating the enhanced cell membrane permeability induced by sonoporation increased EXO-DVDMS uptake. Increased permeability of cell membrane improved the delivery of sono-sensitizer into tumor cell, possibly increasing the anti-cancer efficacy 49. Taken together, the intracellular DVDMS uptake was maximized by exosomal encapsulation plus US.

### *In vitro* cytotoxicity of EXO-DVDMS

**Figure 4:**
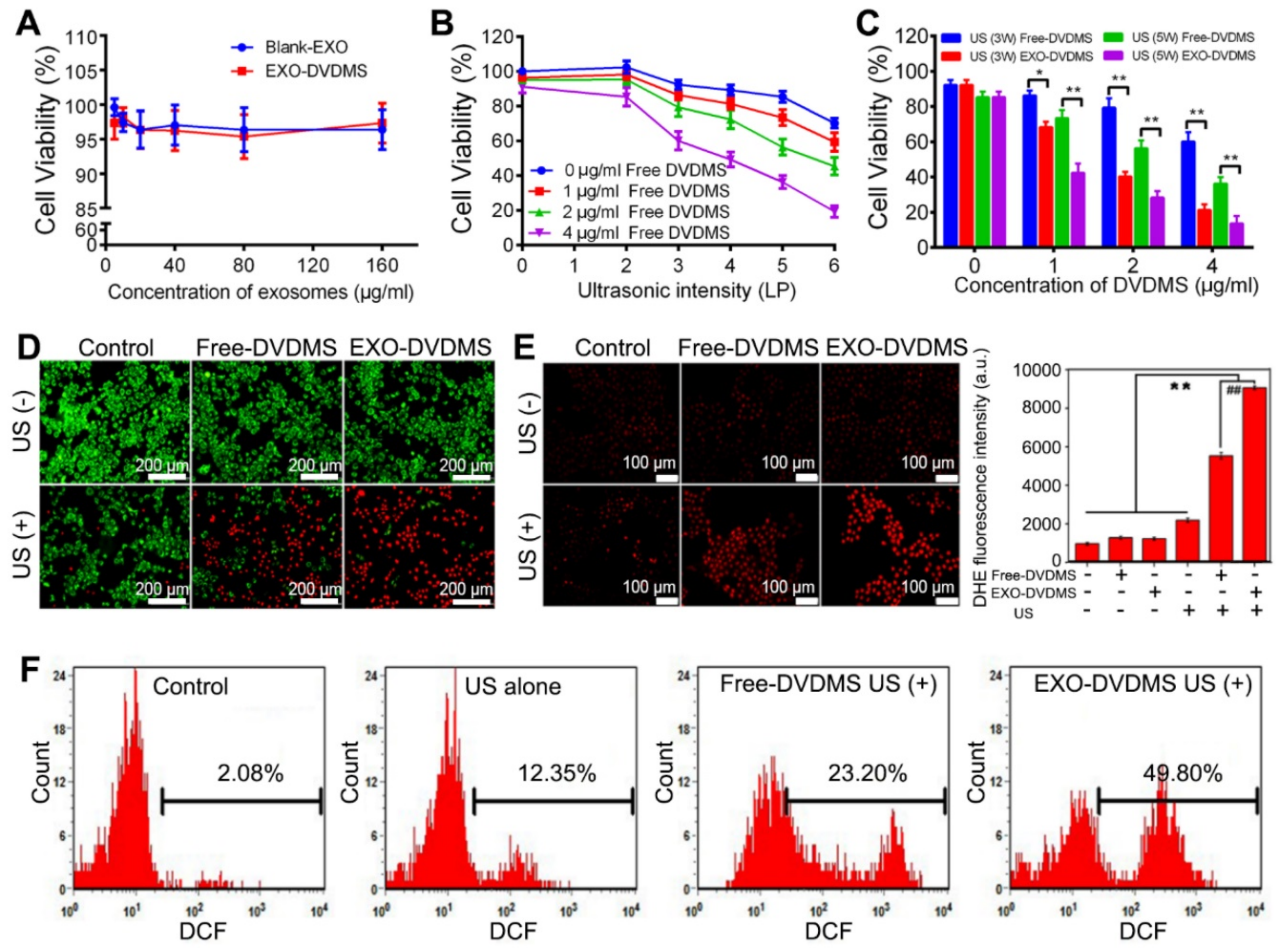
** Analysis of sono-cytotoxicity of EXO-DVDMS. (A)** Cell viability curve of 4T1 cells after 24 h incubation with Blank-EXO and EXO-DVDMS at different exosome concentration (0-160 µg/mL). **(B)** Cell viability curve of 4T1 cells at 24 h after treatment with Free-DVDMS (0-4 µg/mL) plus ultrasound (60 s duration) at various intensity ranging from 0 to 6 W (LP). **(C)** Comparison of cell viability on 4T1 cells by Free-DVDMS and EXO-DVDMS after different US treatment at 3W and 5W (60 s duration). **(D)** The cytotoxicity of Free-DVDMS and EXO-DVDMS mediated SDT measured by calcein AM/PI double staining (3W, 60 s). Viable cells were stained green with calcein-AM, and damaged cells were stained red with PI (Bar=200 µm.). **(E)** Intracellular superoxide anion generation in 4T1 cells detected by DHE at 2 h post treatment using fluorescence microscope, the representative figures (left) and statistic data (right) are shown. **(F)** Intracellular ROS generation detected by DCFH-DA as measured by flow cytometry. *p<0.05, ** p<0.01 between groups. Data shown are mean ± S.D. of three batches.

As determined by the MTT assay, neither blank-EXO nor EXO-DVDMS exhibited any cytotoxicity when incubated with 4T1 cells for 24 h, even at the high concentration of 160 µg/mL, indicating good biocompatibility **(Figure 4A)**. Meanwhile, cell viabilities were 96.33±2.33%, 95.06±2.01%, 91.20±3.60% in Free-DVDMS treated groups at concentrations of 1, 2, 4 µg/mL, respectively **(Figure 4B)**. However, the cell viability was significantly decreased when combined with US, showing the DVDMS-mediated SDT efficacy is highly US/drug-dose dependent **(Figure 4B)**. Under 2W US, both DVDMS-SDT and EXO-DVDMS-SDT did not show significant sonotoxicity **(Figure S10)**. As shown in **Figure 4C**, US alone at 3 W or 5 W slightly affected the cells, with viability as high as 92.30±2.69% and 85.33±3.19%. US plus 1, 2 and 4 µg/mL Free-DVDMS decreased cell viability to 86.35 ± 2.31%, 79.32 ± 5.31% and 60.10 ± 4.19% at 3W, and to 73.36 ± 4.60%, 56.32 ± 3.97% and 36.30 ± 3.70% at 5 W. By contrast, EXO-DVDMS exhibited much stronger sonodynamic cytotoxicity under the same parameters. The cell viability in EXO-DVDMS plus 3W US decreased to 68.32 ± 3.21%, 40.32 ± 2.67%, and 21.30 ± 3.22% with DVDMS at 1, 2 and 4 µg/mL, respectively. Under 5 W US, EXO-DVDMS-SDT further decreased the cell viability to 42.32 ± 5.3%, 28.32 ± 3.70%, 13.60 ± 3.11% at 1, 2 and 4 µg/ml DVDMS. As shown by calcein-AM/PI staining **(Figure 4D)**, cells incubated with 2 µM Free-DVDMS or EXO-DVDMS, or solely exposed to US, did not show higher proportion of dead cells (red signal) compared to the untreated control. When treated with free-DVDMS and US, the percentage of dead cells increased sharply, with even higher rate of cell death in the EXO-DVDMS plus US-treated group. Taken together, EXO-DVDMS exhibited much higher cytotoxicity than Free DVDMS under the same experimental conditions, which could decrease the sensitizer dosage and ultrasound energy, thereby reducing side effects. Therefore, the gentler US, 3W rather than 5W, was preferred *in vivo* to minimize the impact on normal tissues.

### Ultrasonic stimulation and EXO-DVDMS synergistically enhanced intracellular ROS production

Excessive ROS can damage lipids, proteins and DNA, along with causing mitochondrial dysfunction, disruption of ion balance, and loss of membrane integrity 19. Since cell damage induced by SDT is closely related to ROS generation 45, 50, we hypothesize that the enhanced therapeutic efficiency of EXO-DVDMS-SDT is also a result of high ROS levels, including that of superoxide radical anions which are produced inevitably during SDT. Compared to the control, neither free- nor exosomal DVDMS alone significantly affected superoxide generation, as measured by red DHE fluorescence, while US alone (3 W, 60 s) slightly increased the fluorescence intensity **(Figure 4E)**. In contrast, Free-DVDMS plus US significantly increased superoxide generation, while EXO-DVDMS resulted in even brighter DHE fluorescence when combined with US, under the same conditions. Similar results were seen with DCFH-DA staining **(Figure 4F)**. Compared to the control, Free-DVDMS and EXO-DVDMS increased DCF fluorescence intensity to 23.2% (p<0.05) and 49.8% (p<0.001) respectively, when combined with US. Thus, exposure to US and an exosomal encapsulated sonosensitizer markedly increased the oxidative stress in 4T1 cell, which is the likely mechanism of the anti-cancer effects of SDT.

### Subcellular re-localization of exsomal DVDMS under ultrasonic stimulation

**Figure 5:**
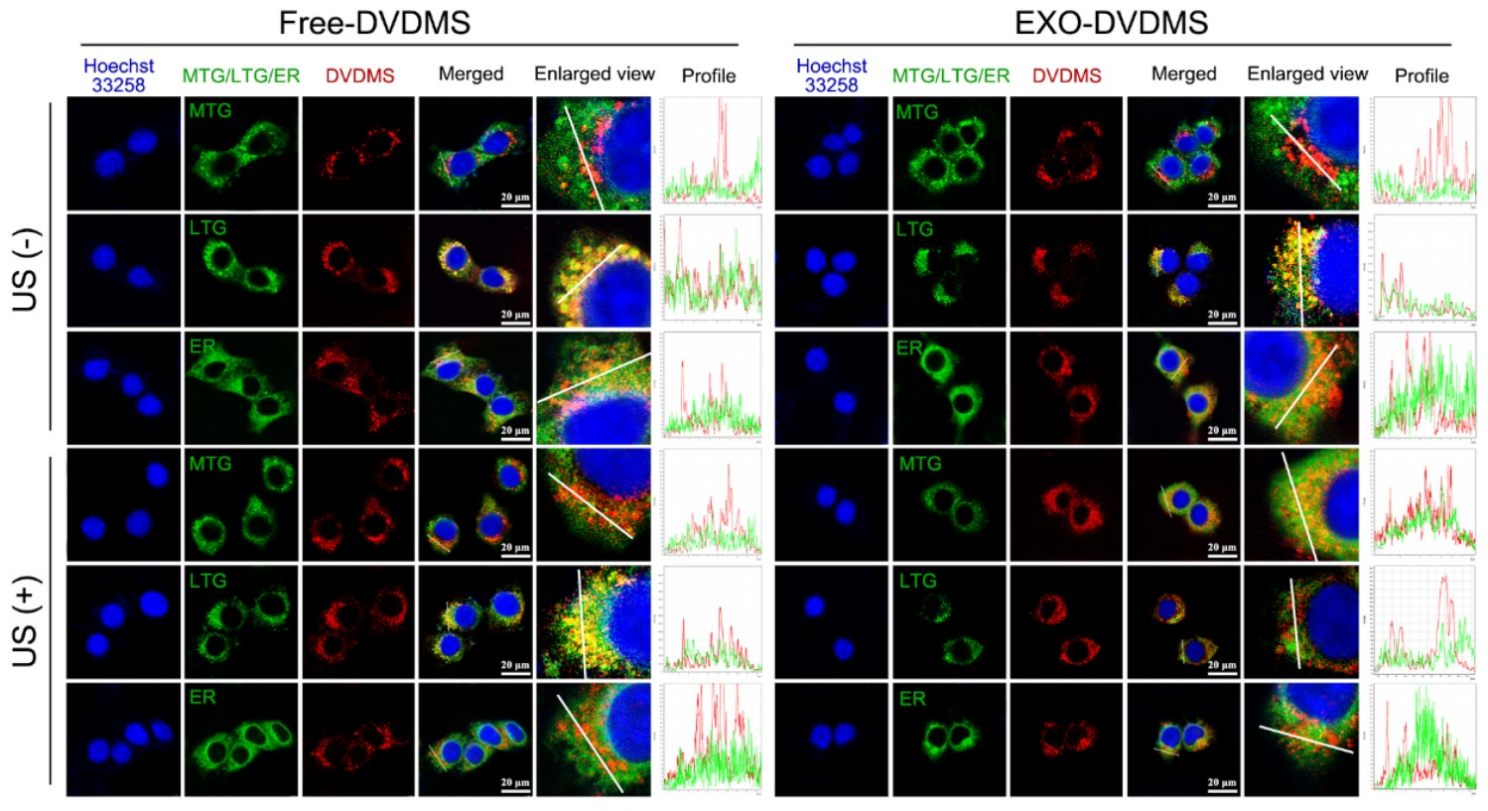
** Subcellular localization of Free-DVDMS and EXO-DVDMS upon ultrasound treatment in physiological condition (with serum).** 4T1 cells were seeded in chamber slides and incubated for 6 h with 5 µg/mL DVDMS in either Free-DVDMS or EXO-DVDMS form, then exposed to ultrasound (2 W, 30 s) prior to confocal imaging. DVDMS fluorescence (red), MTG, LTG and ER fluorescence (green), nuclear DAPI fluorescence (blue) were captured by confocal microscopy, scale bar=20 µm.

The intracellular transport of EXO-DVDMS in 4T1 cells under US stimulation was studied by tracking the subcellular localization of free DVDMS in serum-free and serum-additive conditions, and that of EXO-DVDMS in the presence of serum, following 30 s pulse of 2 W US. Under serum-free with no US, the red fluorescence signals of DVDMS co-localized with the green fluorescence signals of MitoTracker Green (MTG), and partially with the green signals of ER (endoplasmic reticulum) tracker but not with that of LysoTracker Green (LTG), indicating that DVDMS mainly accumulated in the mitochondria **(Figure S11)**. However, in physiological condition with serum, Free-DVDMS largely co-localized with LTG **(Figure 5)**, indicating an altered intracellular route via the lysosomes, due to aggregation of DVDMS and proteins 44. For EXO-DVDMS also, DVDMS was localized in the endo/lysosomes, indicating cellular uptake via endocytosis, consistent with the major cellular internalization mode of exosomes 51. Intracellular Free-DVDMS localization was not affected by US stimulation, while that of EXO-DVDMS shifted from the lysosomes to the mitochondria. This indicates that, on the one hand, EXO-DVDMS-SDT induces lysosomal degradation and endosomal opening to trigger cargo release, and on the other hand, promotes cargo transport and redistribution from endosomal vesicles to the cytoplasm and mitochondria, thereby initiating cell death-signaling pathways and improving the therapeutic efficiency of the cargos.

### *In vivo* bio-distribution and long-term circulation of EXO-DVDMS

Unlike the normal tissues, the tumor vasculature normally lacks an effective lymphatic drainage system and thus allowing the passive targeting of tumors via the EPR effect 35. Exosomes are natural nanomaterials with the right size for EPR, making them highly suitable for targeted drug delivery. The *in vivo* bio-distribution of DiR-labelled EXO-DVDMS (200 µg total protein) in BALB/c tumor-bearing mice was evaluated by a real-time NIR fluorescence imaging system. As shown in **Figure S12A**, a strong fluorescence signal was detected at the tumor site after 24 h, which further intensified by 48 h, then the fluorescence intensity gradually decreased indicating exosomal metabolism.

One of the advantages of using exosome as drug delivery system is that they are naturally protected from degradation in the bloodstream 34. Thus, exosomal encapsulation prevents the premature release of the drug and its recognition by the reticuloendothelial system, and ensures prolonged blood circulation. To assess whether the EXO-DVDMS had a long circulation lifetime, pharmacokinetic studies were also carried out in the BALB/c mouse model. Compared to the free DVDMS, EXO-DVDMS showed a significantly slower clearance, a higher area under the curve (AUC (0-∞)), and a higher elimination half-life (t1/2). As shown in **Figure S12B**, the AUC (0-∞) and t1/2 of EXO-DVDMS were 2.09- and 1.42-fold higher than that of free DVDMS respectively, indicating that exosomal loading significantly elongated the circulation of DVDMS.

### *In vivo* homotypic targeting delivery of EXO-DVDMS

**Figure 6:**
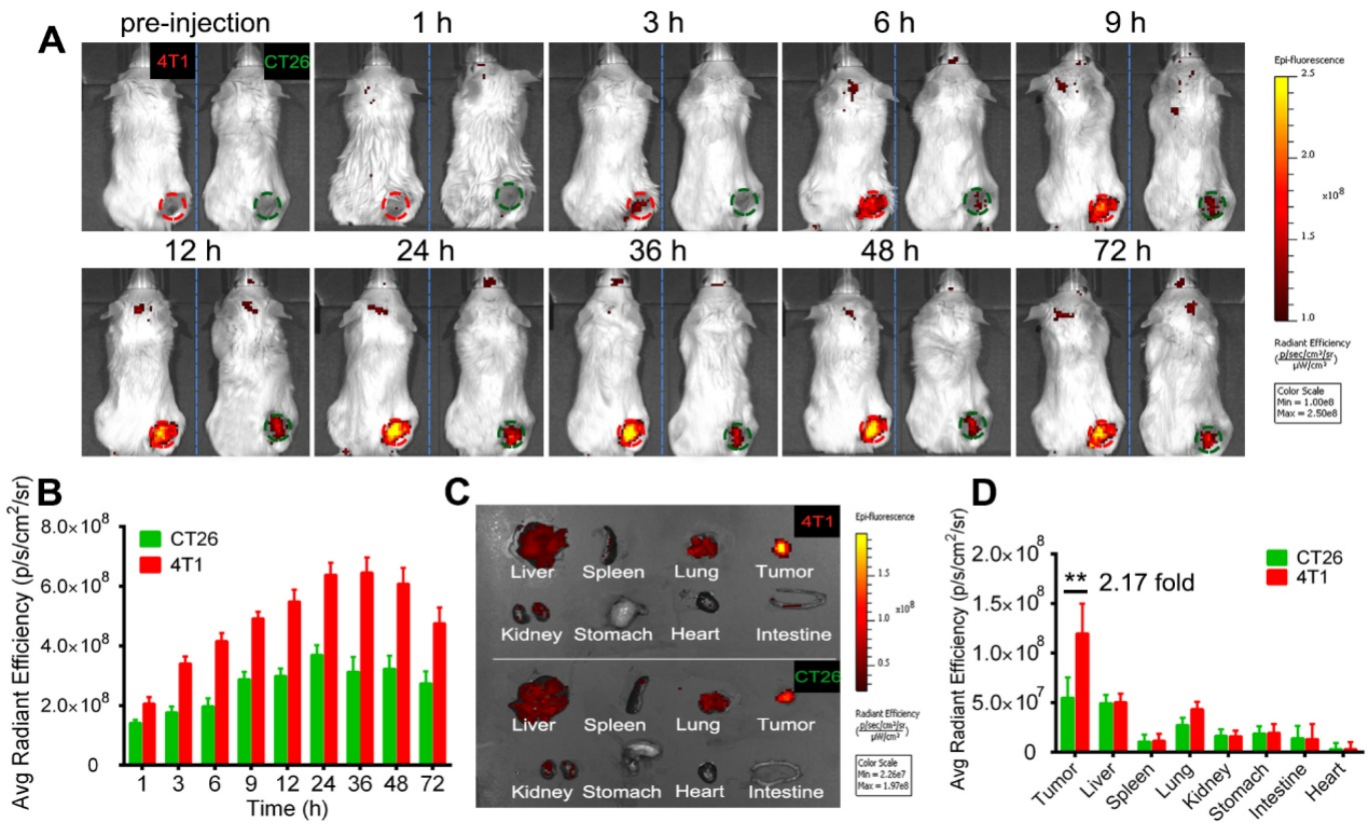
***In vivo* homotypic targeting potential of EXO-DVDMS derived from 4T1 tumor cells**. **(A)** *In vivo* images of subcutaneous 4T1 tumor-bearing (homotypic tumor, red circle) and CT26 tumor-bearing (non-homotypic tumor, green circle) mice after intravenous injection of EXO-DVDMS (2 mg/kg). **(B)** Quantification of EXO-DVDMS in 4T1 and CT26 tumors *in vivo.* **(C)** *Ex vivo* images of tumors and major organs after 24 h of EXO-DVDMS administration. **(D)** Quantitative analysis of fluorescence intensity of tumors and major organs after 24 h of administration (p/s/cm^2^/sr). **p<0.01, between groups. Data shown are mean ± S.D. of three batches.

To investigate the homotypic targeting ability of 4T1 cell derived exosomes, EXO-DVDMS accumulation in 4T1 tumor-bearing (homotypic tumor) and CT26 tumor-bearing (non-homotypic tumor) mouse models was measured. As shown in **Figure 6**, compared to non-homotypic CT26 tumor, EXO-DVDMS preferentially accumulated into homotypic 4T1 tumor at all detected time points. The average radiant efficiency of EXO-DVDMS in 4T1 tumor was about 2.17-fold higher than that in CT26 tumor. To avoid individual differences between mice, a mouse model simultaneously bearing two different types of tumors (CT26 tumor and 4T1 tumor) was established in this study. Similarly, the accumulation of 4T1 cell derived exosomes in 4T1 tumor was about 1.94-fold higher than that in CT26 tumor (**Figure S13),** consistent well with *in vitro* study **(Figure 3B).**

**Figure 7:**
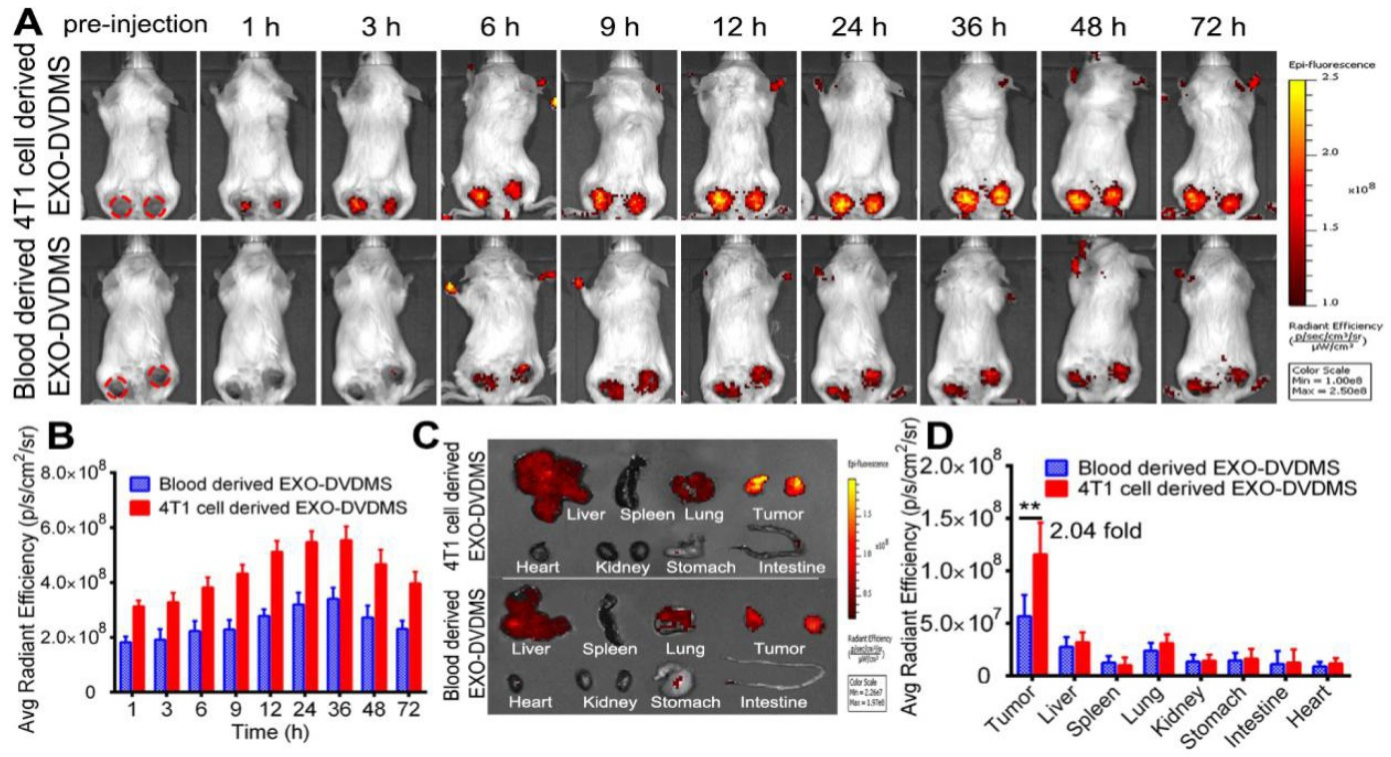
***In vivo* bio-distribution and tumor accumulation of 4T1 cell derived EXO-DVDMS and blood derived EXO-DVDMS**. **(A)** *In vivo* images of subcutaneous dual-tumor bearing mice after intravenous injection of 4T1 cell derived EXO-DVDMS and blood derived EXO-DVDMS (2 mg/kg). **(B)** Quantification of (A)*.* **(C)** *Ex vivo* images of tumors and major organs after 24 h of 4T1 cell derived EXO-DVDMS and blood derived EXO-DVDMS administration. **(D)** Quantitative analysis of fluorescence intensity of tumors and major organs after 24 h of administration (p/s/cm^2^/sr). **p<0.01, between groups. Data shown are mean ± S.D. of three batches.

Besides, we prepared blood derived EXO-DVDMS and compared the tumor accumulation of homotypic 4T1 cell derived EXO-DVDMS (4T1-EXO-DVDMS) and non-homotypic blood derived EXO-DVDMS (B-EXO-DVDMS) in 4T1 tumor bearing mice after 2 mg/kg 4T1-EXO-DVDMS/B-EXO-DVDMS injection. As shown in **Figure 7**, 4T1-EXO-DVDMS displayed much higher DVDMS fluorescence (2.04-fold) in homotypic tumors compared with that of B-EXO-DVDMS. In summary, the *in vivo* homologous targeting of EXO-DVDMS were verified in the present study.

### Ultrasound guided EXO-DVDMS accumulation and permeation in exposed tumors

**Figure 8:**
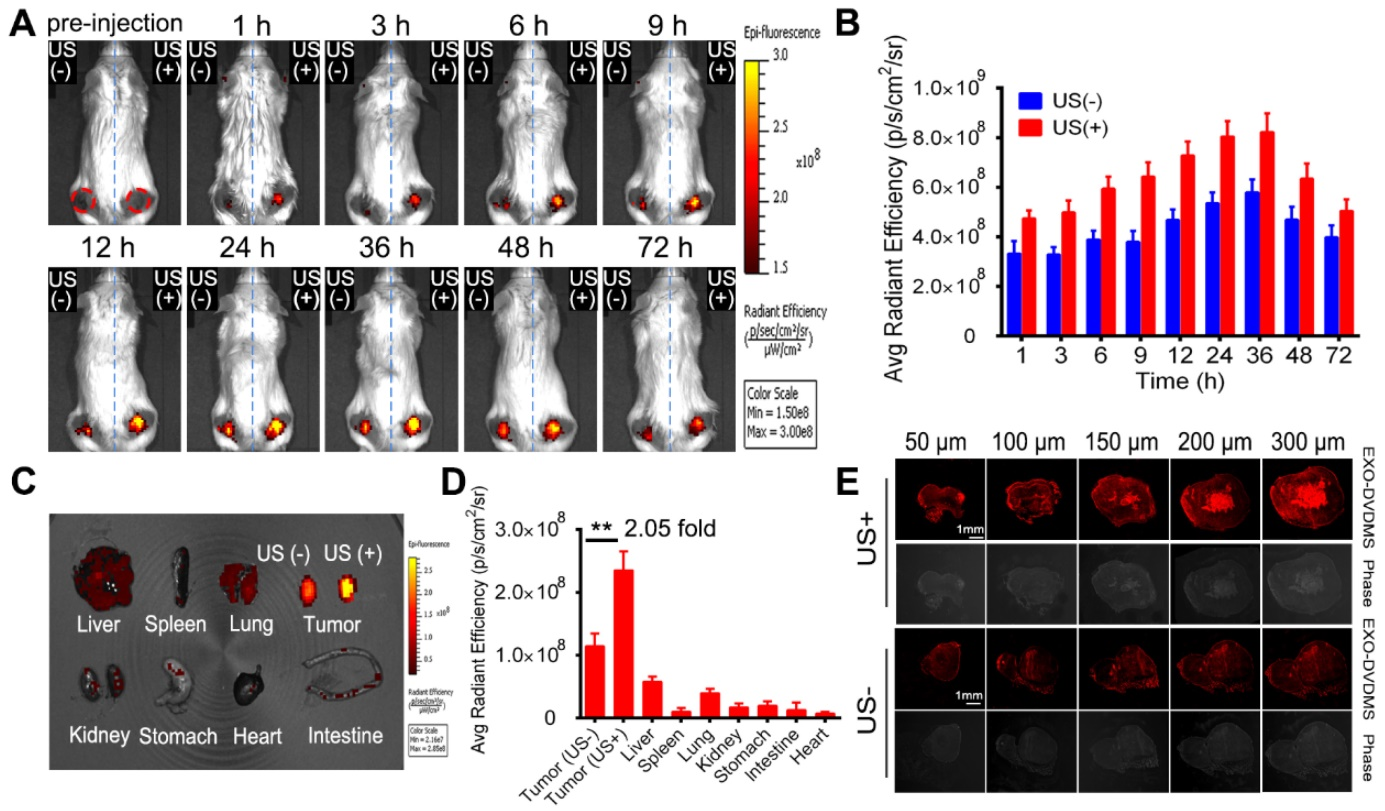
***In vivo* bio-distribution and ultrasound promoted *in situ* EXO-DVDMS deposition and permeation**. **(A)** *In vivo* images of subcutaneous dual-tumor bearing mice with or without US after intravenous injection of EXO-DVDMS (2 mg/kg). Right tumors were exposed to US1 (2 W, 3 min), left tumors without US1 were used for comparison. **(B)** Quantification of EXO-DVDMS in 4T1 tumors *in vivo.* **(C)** *Ex vivo* images of tumors and major organs after 24 h of administration. **(D)** Quantitative analysis of fluorescence intensity of tumors and major organs after 24 h of administration (p/s/cm^2^/sr).** (E)** US1-assisted drug delivery and penetration after injection with EXO-DVDMS. **p<0.01, between groups. Data shown are mean ± S.D. of three batches.

The most important pharmacokinetic principle for the design of macromolecular drugs or nanomedicines is the EPR, which was first termed by Maeda in 1986 52. Despite the initial promise of the approved nano-formulations, the outcomes of clinical trials are unsatisfactory 53. The conventional nanodrugs indeed alleviate some dose-limiting toxicities, however, they cannot improve the tumor uptake efficiency in patients 54, 55. In fact, the unsatisfied drug accumulation is due to the EPR effect varies depending on a patient's pathological and physiological characteristics and clinical condition. Moreover, multiple biological and pathophysiological barriers also withhold the accumulation of nanoparticles, causing a heterogeneous extravasation and drug distribution. To overcome such tricky problems, several feasible strategies were developed to augment the EPR effect. Extracorporeal low-intensity ultrasound has shown great potentials in drug delivery and therapeutics 56. Preliminary studies show that US sonoporation plays a role in improving drug delivery into solid tumors by enhancing vascular permeability 57. US cavitational effects, such as jet formation, shock waves, and microstreaming, could also increase cell membrane permeabilization, produce a reversible opening of tight junctions in the vascular endothelium and enhance the accumulation and permeation of nanoparticles 57. Hence, in this study, US1 (guided-ultrasound at 2 W for 3 min) plus SonoVue® were applied immediately after injection of EXO-DVDMS to increase the particle deposit at the target tumor site. Considering the biodistributions of DVDMS and exosome were not always the same after systematically injection of EXO-DVDMS due to the possibility that DVDMS may prematurely release before reaching the tumor sites, the distributions of exosomes (labeled by DiR) and EXO-DVDMS were respectively investigated. As shown in **Figure 8 A and B**, the fluorescence signal increased gradually after intravenous injection of EXO-DVDMS combined with US1, and peaked approximately 24-36 h after injection. The DVDMS fluorescence signals in the right tumor (+US) were about 2.05-fold higher than that of left tumor (-US), indicating that selective irradiation by focused US could promote nanoporphyrins accumulation at the specific site **(Figure 8 C and D)**. The results from exosomes (labeled by DiR) consist with that of EXO-DVDMS, suggesting good stability of EXO-DVDMS *in vivo* (**Figure S12C-E**).

The vessel-wall structure in tumors is abnormal with wide inter-endothelial junctions and irregularly thick or thin basement membrane 35, which makes the vasculature “leaky” and hyperpermeable at some places. This not only limits drug penetration into tumors but also causes uneven distribution of drugs within the tumor tissues 58. Therefore, we further examined the distribution of EXO-DVDMS *in situ* under US stimulus. As shown in **Figure 8E**, much stronger and diffused DVDMS red fluorescence was observed throughout the stimulated tumor, indicating that US promoted the penetration and intratumoral diffusion of EXO-DVDMS. The results from DiO labeled EXO-DVDMS coincided with the above phenomenon (**Figure S12F**).

Moreover, the *in vivo* bio-distribution and imaging ability of EXO-DVDMS were also compared with Free-DVDMS. As shown in **Figure S14 A and B**, fluorescence signal in tumor region increased quickly, reached a peak value at 3 h, and decreased gradually after 6 h in Free-DVDMS group, which was in accord with our previous study 59. For EXO-DVDMS, fluorescence signal peaked at 24-36 h after injection and sustained high level until 72 h. The organ/tissue-specific distribution of EXO-DVDMS and Free-DVDMS was examined at 24 h post injection. The average radiant efficiency of tumor in EXO-DVDMS group was about 3.22-fold greater than that of Free-DVDMS group (**Figure S14 C and D).** Undoubtedly, US1 stimulation increased much higher EXO-DVDMS accumulation *in situ* than that of Free-DVDMS. These results suggest that EXO-DVDMS greatly improved the *in vivo* imaging ability, bio-distribution and tumor accumulation of DVDMS.

### Ultrasound triggered SDT with EXO-DVDMS significantly inhibited tumor growth

**Figure 9:**
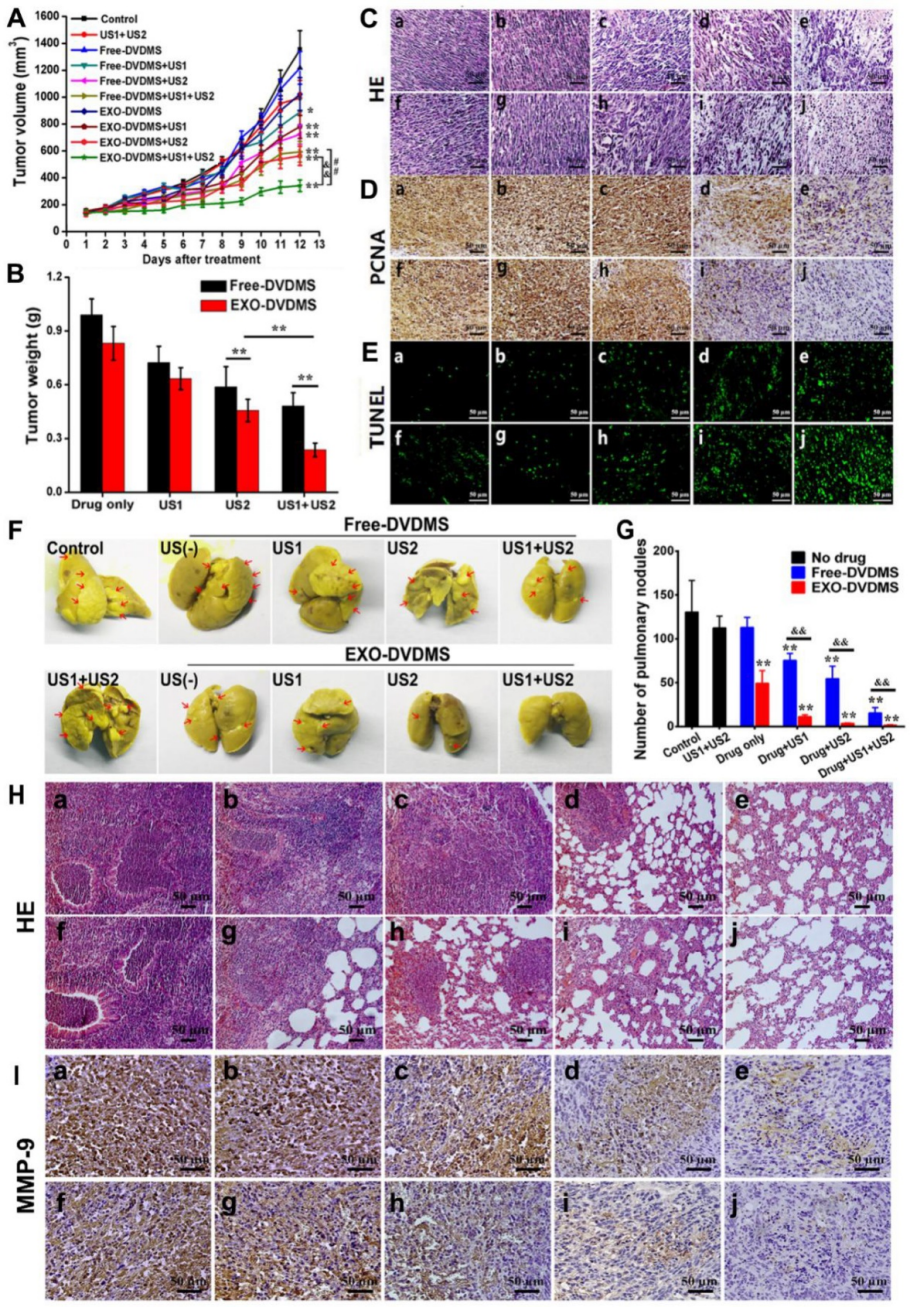
***In vivo* enhanced therapeutic effect and lung metastasis suppression of EXO-DVDMS-SDT in 4T1-xenograft mice. (A)** Tumor growth curves of different groups of 4T1 tumor-bearing mice (n=6). **p<0.01 versus control, ## p<0.01 versus Free-DVDMS+US1+US2, && p<0.01 versus EXO-DVDMS+US2. **(B)** Tumor weight inhibition ratio at the 12^th^ day of different treatment groups. **p<0.01 between different groups. **(C)** H&E stained images of tumor sections collected from different treated groups of mice. Bar=50 µm.** (D)** Immunohistochemistry detection for PCNA after different treatments. Bar=50 µm. **(E)** *In situ* apoptosis by TUNEL assay. Bar=50 µm. **(F)** Photos of lungs after soaking in Bouin's solution showing spontaneous pulmonary breast cancer metastases (red arrows). The mouse lungs were taken at the 12^th^ post different treatments and the gross appearance of pulmonary nodules was photographed. **(G)** The pulmonary nodules were manually counted and the average numbers were calculated in different groups. ** p<0.01 versus control, && p<0.01 EXO-DVDMS versus Free-DVDMS. Data shown are mean ± S.D. of five batches. **(H)** H&E stained images of lung sections collected from different treated groups of mice. Bar=50 µm. **(I)** Immunohistochemistry detection for MMP-9 level of 4T1 tumors after different treatments. (a) Control, (b) US1 (2 W, 3 min)+US2 (3 W, 3 min), (c) Free-DVDMS (2 mg/kg), (d) EXO-DVDMS (2 mg/kg), (e) Free-DVDMS+US1, (f) EXO-DVDMS+US1, (g) Free-DVDMS+US2, (h) EXO-DVDMS+US2, (i) Free-DVDMS+US1+US2, (j) EXO-DVDMS+US1+US2.

As shown in **Figure 9**, the *in vivo* antitumor efficacy of EXO-DVDMS and Free-DVDMS was subsequently investigated by using a guiding ultrasound (US1, 2 W, 3 min) and a therapeutic ultrasound (US2, 3 W, 3 min) at different time windows post injections, and the tumors were photographed after 12 days for direct observation (**Figure S15**). The percentage inhibition of tumor volume following different treatments are shown in **Figure 9A**. Neither free nor exosomal DVDMS (2 mg/kg) inhibited tumor growth in the absence of US, while stimulation with US1+US2 alone slightly inhibited tumor growth (26.63%). A single therapeutic ultrasound (US2) combined with Free-DVDMS or EXO-DVDMS on the other hand markedly inhibited tumor growth in terms of both volume and weight (46.59% and 58.45% respectively; p < 0.01 vs. control). As expected, US1+US2 along with Free-DVDMS and EXO-DVDMS resulted in maximum tumor growth inhibition (56.20 % and 74.88 % respectively; p < 0.001 vs. control, p < 0.01 for Free-DVDMS vs. EXO-DVDMS), indicating the crucial role of the guiding ultrasound which markedly strengthened the antitumor effect by increasing accumulation and permeation of the nanosensitizers. In addition, the tumor size in EXO-DVDMS-SDT group was much smaller than that of the Free-DVDMS-SDT group. The superior antitumor efficiency of the former may be due to the longer circulation ability, good bioavailability, high ROS yield and the spatiotemporally controlled US activation. On the 12^th^ day of treatment, the mice were sacrificed and the tumors were weighed, which showed similar trend as tumor volume inhibition **(Figure 9B)**.

Histological examination indicated more extensive tumor tissue damage in the US1+US2 plus EXO-DVDMS treated group compared to US2 plus EXO-DVDMS and US1+US2 plus Free-DVDMS groups **(Figure 9C)**. Furthermore, primary antibody against proliferating cell nuclear antigen (PCNA) level declined significantly **(Figure 9D)** and TUNEL staining increased markedly **(Figure 9E)** after US1+US2 plus EXO-DVDMS treatment compared to the other treatment modalities, indicating inhibition of tumor cell proliferation and apoptosis induction following EXO-DVDMS-SDT.

### EXO-DVDMS-SDT effectively inhibited breast cancer-lung metastasis

Cancer metastasis is associated with poor prognosis and is the foremost cause of patient death. The development of robust therapeutic drugs that interrupt the primary causes of metastasis is a daunting but important challenge 60. An innovative work shows that the artificial pre-metastatic niche of tumor extracellular vesicles in a 3D scaffold was able to capture cancer cells, resulting in strikingly increased survival of the animal 61. Likewise, the intrinsic features of tumor derived exosomes endow them with the capacity to target their parental tumors and trace pre-metastatic niches, this may help EXO-DVDMS-SDT destroy the potential pre-metastatic niches or affect their formation. Herein, we analyzed the anti-metastatic effects of EXO-DVDMS-SDT using the lung metastasis model. As shown in **Figure 9F and G**, lungs of the untreated control mice, and that of the mice receiving ultrasound or Free-DVDMS monotherapies displayed multiple surface metastatic nodules, with the respective average number of nodules being 130.5 ± 36.06, 112.5 ± 13.43 and 113.0 ± 11.31. While Free-DVDMS+US1, Free-DVDMS+US2 and Free-DVDMS+US1+US2 reduced lung metastases to some extent (the respective average number of pulmonary nodules were 75.5 ± 7.78, 54.67 ± 14.05 and 15.5 ± 6.20), the same ultrasonic modalities with EXO-DVDMS drastically decreased the average number of pulmonary nodules to 11.33 ± 2.08, 3.5 ± 0.71 and 1.5 ± 0.71 respectively. Furthermore, the lung surfaces of the EXO-DVDMS+US1+US2 treated mice were very similar to that of the normal lung. Histological analyses of lung tissues receiving the different treatments further confirmed the superior anti-metastatic effect of EXO-DVDMS+US1+US2 **(Figure 9H)**. Matrix metalloproteinases (MMPs) are highly expressed in tumor cells and play important roles in tumor invasion, metastasis and angiogenesis.

The expression level of MMP-9 declined significantly in US1+US2 plus EXO-DVDMS treated group compared to the others **(Figure 9I)**, which is consistent with the anti-metastatic effect of EXO-DVDMS+US1+US2. Interestingly, EXO-DVDMS monotherapy significantly decreased the average number of pulmonary nodules to 49.25 ± 14.52. Considering that EXO-DVDMS alone did not show any significant anti-tumor effects, factors other than tumor growth inhibition may help reduce lung metastases in this group. Previous study has found that cancer cells release biological “drones”, called exosomes, circulating in the blood and armed with the protein PD-L1, which cause T cells to tire before they have a chance to reach the tumor and do battle 62. This implies the possible pro-metastatic and immunosuppressive effects of tumor cell derived exosomes. Interestingly, Andrew Riches et al. have shown a feedback regulatory pathway of exosome concentrations by exosomes in the extracellular environment derived from their own cells, in which the increasing concentration of homotypic exosomes would inhibit exosome secretion from tumor cells 63. Based on these, we surmised the obtained result was likely due to the high-level accumulation of EXO-DVDMS (with the cloak of tumor derived exosomes) in the tumor tissue, which probably down-regulated the exosome release from tumors and thus decreased the pro-metastatic and immunosuppressive effects of tumor derived exosomes.

### *In vivo* biocompatibility assessment

To assess the biocompatibility of EXO-DVDMS-SDT *in vivo*, body weight of tumor-bearing mice was monitored during the treatment process, and no major changes were observed for different groups (**Figure S16A**). Histological examination of the major organs (heart, liver, spleen, and kidney) showed negligible damages, indicating good biosafety (**Figure S16E**). Furthermore, liver/kidney function indices [aspartate aminotransferase (AST)/alanine aminotransferase (ALT), blood urea nitrogen (BUN) levels] also showed no obvious changes compared with the controls (**Figure S16 B, C and D**). These results demonstrate that EXO-DVDMS has good biocompatibility and could be considered as an effective drug delivery platform.

In summary, a smart endogenous nano-sonosensitizer, EXO-DVDMS, with the “cloak” of homotypic tumor exosome, was successfully prepared and applied for enhanced SDT. EXO-DVDMS with homo-targeting ability exhibited enhanced preferential tumor accumulation and deep penetration under the guidance of ultrasound. In response of US stimulation and low pH in tumor, EXO-DVDMS achieved excellently controlled drug release, subsequently initiated an enhanced sonodynamic reaction under therapeutic US. Compared to Free-DVDMS, EXO-DVDMS provides the following attractive advantages. (i) Improved physiological drug stability in acid environment and enhanced ROS yield; (ii) Intrinsic targeting ability of homotypic exosome allowing better pharmacokinetics and optimal biodistribution; (iii) Capability to serve as spatiotemporally controlled platform for multi-drug delivery; (iv) Superior anti-metastatic activity and satisfied biosafety. However, there are still several obstacles need to be solved in the way of clinical translation of exosome based nano-sonosensitizers such as (i) quick and low-cost preparation of large-scale cell derived exosomes, (ii) new tumor models with high heterogeneity approaching to clinic practice. In addition, the anti-tumor outcomes of Free-DVDMS-SDT/EXO-DVDMS-SDT under multiple therapeutic US treatment, and the long-term inhibitory effects as well as tumor recurrence are worthy of deep investigations in the near future.

## Conclusion

We designed an ultrasound-responsive natural exosome-based delivery system for DVDMS, a potent transforming sonosensitizer. This system protected the acidic instability of free DVDMS and by using sequential ultrasound stimulation, further helped in targeted accumulation, deep tissue penetration, and sustained DVDMS release *in situ*, resulting in highly effective tumor-specific SDT, as manifested by decreased tumor-proliferation and increased apoptosis-induction, along with excellent biocompatibility. In addition, the combined use of a targeting and therapeutic US plus EXO-DVDMS drastically inhibited lung metastasis. The possible underlying mechanisms of EXO-DVDMS-SDT were ROS generation, cavitation and increased membrane permeability. Our sono-responsive nano-delivery system provides novel insights into the application of natural vesicles as intelligent 'cruise missile' for cancer precision theranostics.

## Materials and Methods

### Materials

DVDMS (molecular formula: C_68_H_66_N_8_O_9_Na_4_, molecular weight: 1,230.265 Da, purity: >98%) was the property of Qinglong Hi-tech Co. Ltd. (Jiangxi, China). DVDMS was dissolved in phosphate-buffered saline (PBS) to a stock concentration of 2 mg/mL and stored in the dark at -20 °C.

3-(4, 5-Dimethylthiazol-2-yl)-2, 5-diphenyltertrazolium bromide tetrazolium (MTT), singlet oxygen sensor green (SOSG), terephthalic acid (TA), fluorescein isothiocyanate-dextran (FD500), 2,7-dichlorodihydrofluorescein diacetate (DCFH-DA), Dihydroethidium (DHE), Calcein-AM/propidium iodide (PI), Hoechst 33258, the fluorescent dyes 3,3-Dioctadecyloxacarbocyanine perchlorate (DiO), 1,1-dioctadecyl-3,3,3,3-tetramethylindotricarbocyaine iodide (DiR), mitochondrion tracker green (MTG), lysosome tracker green (LTG) and endoplasmic reticulum tracker green (ER) were purchased from Invitrogen (Thermo Scientific Inc., USA). Primary antibodies against proliferating cell nuclear antigen (PCNA) and matrix metalloproteinase-9 (MMP-9), were purchased from Abcam (Cambridge, UK). *In situ* cell death detection (terminal deoxynucleotidyl transferase-mediated dUTP nick-end labeling, TUNEL) kit was purchased from Roche. Test kits of serum alanine aminotransferase (ALT), aspartate aminotransferase (AST), and urea nitrogen (BUN) were purchased from Nanjing Jiancheng Bioengineering Institute (Nanjing, China).

### Cell culture and animal model

Mouse mammary cancer 4T1 cells, human breast cancer cell lines MDA-MB-231 and MCF-7, mouse colon carcinoma CT26 cells and mouse embryo fibroblast NIH/3T3 cells were obtained from the Cell Resource Center of the Chinese Academy of Science, China. 4T1 cells, MDA-MB-231 cells and MCF-7 cells were cultured in Dulbecco's modified Eagle's medium (DMEM, Gibco, Life Technologies, Inc., USA), and CT26 cells and NIH/3T3 cells were cultured in RPMI 1640 medium (Sigma-Aldrich). All cells were supplemented with 10% FBS (Hyclone, USA), 100 U/mL penicillin, 100 µg/mL streptomycin and 1mM L-glutamine, and cultured in an incubator with 5% CO_2_ and 100% humidity at 37°C. FBS was ultra-centrifuged for 18 h at 120,000×g to remove contaminating exosomes. All media was filtered with 0.22 µm low protein binding cellulose acetate filter from Thermo Fisher Scientific, Inc. (Rockford, IL) before use. Cells in the exponential phase of growth were used in each experiment.

The BALB/c mice (female, 18-20 g body weight) were supplied by the Experimental Animal Center of Fourth Military Medical University (Xi'an, China) and housed at room temperature with a 12 h light/dark cycle and allowed free access to food and water. After 1 week's acclimation, BALB/c mice were subcutaneously injected at the right flanks with 0.1 mL 4T1 cells (1 × 10^7^ cells/mL). When the tumor reached a size of 60-70 mm^3^, the tumor-bearing mice were randomly assigned to different groups and ready for experiment. The animal experiments were performed in accordance with the National Institute of Health's Guide for the Care and Use of Laboratory Animals and were approved by the Institutional Animal Care and Use Committee of Shaanxi Normal University (Xi'an, China).

### Preparation and characterization of EXO-DVDMS

#### Isolation and drug loading of exosomes

Exosomes were isolated from the supernatant of 4T1 cell line. Briefly, cells were grown to 50% confluency in 75T flasks, before the media was replaced by exosome-free DMEM 10% FBS media. After 24 h, the supernatant was collected and purified by a series of centrifugation steps: 10 min 300 ×g, 20 min 1000 ×g and 30 min 10,000 ×g. After removal of cell debris and large vesicles, the supernatant was filtered through 0.2 µm membrane filters. Then, the specially-made exosome isolation reagent based on previous studies with some modifications was added to the filtrate at ratio of 1:4 w/w (reagent: culture media) 40, 41. Culture media/reagent was mixed well by vortexing till a homogenous solution was obtained. The samples were incubated overnight at 4°C, then centrifuged at 6500 × g for 25 min at 4°C. The pellet containing the exosomes was re-suspended in PBS, and stored at -80°C prior to use. The recovery of exosomes was estimated by measuring the protein concentration using BCA method, and validated by western blotting using anti-CD63 and anti-CD9 antibodies (Santa Cruz Biotechnology, USA).

DVDMS with double porphyrin rings were loaded onto the exosomes via a very mild incubation in order to avoid any changes to the surface structure. Briefly, DVDMS and 1 µg/µL exosomes were mixed in different ratios (1:30, 1:15.5, 1:7.5, 1:3), and incubated at room temperature for 30 min. The isolation reagent was then added at ratio of 1:4 w/w and incubated at 4°C for 2 h, followed by centrifugation at 6500 g for 25 min. The supernatant containing the unencapsulated free DVDMS was removed, and the EXO-DVDMS precipitates were re-suspended in PBS. The EE % and DL % of the EXO-DVDMS were calculated after lysing with 0.5% Triton X-100 (EE % = (W*encap-_DVDMS_*/W*total-_DVDMS_*) × 100%, DL % = (W*encap-_DVDMS_* /W*total-_DVDMS_ +*W*exosome*)), and their concentration was determined using fluorescent spectrophotometry (λex = 410 nm, λem = 627 nm).

#### Characterization of EXO-DVDMS

The EXO-DVDMS particles were characterized by TEM after depositing the samples on carbon-coated copper grids and staining with 2% phosphotungstic acid solution (pH=7.4). The exosome morphology was observed at 80 KV using the HT-7700 (Hitachi) microscope. The particle size, zeta potential, and PDI of various exosomal formulations were measured by NTA using ZetaView® (Particle Metrix, Germany). To analyze the spectral properties of different DVDMS formulations, the absorption and emission spectra of Free-DVDMS and EXO-DVDMS were measured with a resolution of 1 nm, in PBS or 50% serum (pH=7.4 or 5.0) to simulate physiological conditions, using a fluorescence spectrophotometer (LS-55, Perkin Elmer Company, USA). In addition, the time-dependent colloidal stability of blank-exosome and EXO-DVDMS in conditioned DMEM containing 10%, 20% and 50 % FBS at 4°C or 37°C were evaluated after 0, 2, 4, 6, 12 and 24 h of incubation by dynamic light scattering (DLS) at 25°C using Delsa Nano C Size/Zeta Potential Analysis Instrument (Beckman Instruments, German).

### Ultrasound exposure system

The ultrasonic device used in this experiment was similar to that described in previous studies 64. The ultrasound exposure system consisted of a power amplifier (AG1020, T&C power conversion Inc., USA), and a focused transducer with frequency of 1.0 MHz and a focal length about 5 cm (ndtXducer®, Northborough, MA). The transducer was of piezoelectric composite and had matching layers and a thick backing. The transducer was submerged in an acrylic tank filled with distilled, degassed water.

In this study, low intensity focused ultrasound (LIFU) was applied to promote drug delivery and trigger SDT 65. For sonication *in vitro*, samples were exposed to US for 60 s duration with load power (LP) of 1-6 W, respectively. To investigate the drug release as a function of exposure time, the exosomes were exposed to US for 30, 60, 90 s at 1-3 W. The temperature of the samples was measured before and immediately after each US exposure using a digital thermometer. For *in vivo* experiments, under anesthesia with 5% chloral hydrate, the tumor region was rightly placed in the focus center of the transducer with the assistant of a multilayer interface-material which improved the acoustic efficiency delivering into the tumor. A guided-ultrasound (US1 at 2 W for 3 min) plus SonoVue® (a contrast agent to enhance US cavitation, Bracco, Italy) were applied immediately after injection of EXO-DVDMS to increase the accumulation of the vesicles at the target site, according to our previous study 48. When EXO-DVDMS reached peak in tumor site (24 h after injection), the tumors were stimulated with US2 (therapeutic ultrasound at 3 W for 3 min) to trigger DVDMS release from the exosomes and initiate a sonodynamic reaction.

### Detection of singlet oxygen

To detect the singlet oxygen generation by DVDMS, singlet oxygen sensor green (SOSG) was used as an indicator 37, 38. Briefly, Free-DVDMS and EXO-DVDMS were diluted in PBS at neutral pH (pH=7.4) or acidic pH (pH=5.0) respectively and placed for 12 h or 24 h to simulate the experimental conditions *in vivo*. After distinct incubation, the Free-DVDMS/EXO-DVDMS was mixed with newly prepared SOSG solution (5.0 µM) and then subjected to US exposure (3 W, 60 s). After sonication, fluorescence emission intensity of SOSG at 525 nm was measured with LS-55 fluorescence spectrophotometer (Enspire, PE, USA) under 504 nm excitation.

### Ultrasound-triggered DVDMS release from EXO-DVDMS

The release of DVDMS from EXO-DVDMS in simulated physiological environment following US stimulation (LP: 1-3W, irradiation time: 30-90s) was evaluated using the dialysis method. Briefly, a thin-walled polypropylene tube containing EXO-DVDMS (in different pH condition/ with or without serum) was placed in the focal zone of US transducer. After stimulation, all samples were placed into dialysis bags (MW 3,000) with 200 mL dialysis buffer, and 500 µL aliquots were collected at different time points and completely ruptured by Triton X-100. The concentration of DVDMS was determined by fluorescent spectrophotometry. The cumulative released DVDMS fraction (CRF%) was calculated as (F_D_/F_0_) × 100%, where F_0_ is the initial fluorescence intensity of DVDMS in a non-stimulated sample, and F_D_ is the fluorescence intensity of cumulative released DVDMS in a stimulated sample at a particular time point.

### Intracellular dissociation of DVDMS from exosomes under ultrasound exposure

The intracellular dissociation of DVDMS from composite EXO-DVDMS under ultrasonic stimulation in 4T1 cells was tracked using laser scanning confocal microscope (Model TCS SP8, Leica, Germany). The 4T1 cells were seeded in an eight-chamber slide with 2×10^4^ cells per well and cultured for 24 h. The cells were then incubated with DiO labelled EXO-DVDMS for 6 h, and subjected to ultrasound (3 W, 60 s). At 15 min after stimulation, the cells were washed thrice with PBS and fixed in 4% paraformaldehyde for 10 min. The cells were stained with Hoechst 33258 for 15 min, and observed under a laser scanning confocal microscope and analyzed by LAS AF Lite 3.3.0 software (Leica, Germany).

### Evaluation of ultrasound cavitation

Acoustic cavitation was evaluated using TA (terephthalic acid) method 64. TA solution (1 mM) as dosimeter solution reacted with hydroxyl radicals formed during ultrasound irradiation in the present of Free-DVDMS or EXO-DVDMS in dark. Immediately after sonication (3 W, 60 s), the fluorescent product 2-hydroxyterephthalic acid (HTA) was measured using a fluorescence photometer (LS-55, PE, USA) at 426 nm.

### *In vitro* cellular uptake of Free-DVDMS and EXO-DVDMS

The time-dependent cellular uptake of blank exosomes and EXO-DVDMS in 4T1 cells was tracked by staining with the lipophilic DiO tracker. Briefly, 4T1 cells were seeded at the density of 2 × 10^5^ cells/mL in 35-mm dishes and cultured overnight. DiO labeled exosomes (75 µg/mL and 150 µg/mL total protein) were added to the cells, and after incubating for 3, 6, 12 and 24 h, the cells were washed and observed under laser scanning confocal microscope. Exosome uptake was also quantified by measuring the intracellular DiO fluorescence intensity using flow cytometry (NovoCyteTM, ACEA Biosciences Inc., CA&USA).

Simultaneously, the cellular uptake of EXO-DVDMS was also analyzed based on the fluorescence of DVDMS at 3, 6, 12, 24 h after incubation using laser scanning confocal microscope. To compare the cellular uptake of Free-DVDMS and EXO-DVDMS in 4T1 cells quantitatively, Free-DVDMS and EXO-DVDMS (DVDMS = 1 µg/mL) were added to the cells. At 12 h after treatment, cells were collected and washed thrice with PBS. The cellular DVDMS fluorescence intensity was quantified by flow cytometry. For ultrasound exposure (2 W, 60 s), samples were collected at 30 min post treatment and subjected to fluorescence imaging (E-600, Nikon, Japan) and quantitative analysis.

To further evaluate the subcellular distributions, Free-DVDMS in serum-free or serum media, and EXO-DVDMS in serum media under 2 W ultrasound in 4T1 cells was investigate. Cells seeded in an eight-chamber slide with 2×10^4^ cells per well and cultured for 12 h. Then cells were incubated with equal DVDMS (5 µg/mL) for 6 h. After that, cells were washed with PBS and exposed to US. At 1 h, MTG, LTG and ER tracker green were added to label the mitochondria, lysosomes and endoplasmic reticulum, respectively. Images were obtained using laser scanning confocal microscope.

### Homologous targeting of EXO-DVDMS *in vitro*

To validate the homotypic specificity of EXO-DVDMS *in vitro*, flow cytometry was applied to measure the cellular uptake of EXO-DVDMS in 4T1 cells, MDA-MB-231 cells, MCF-7 cells, CT26 cells and NIH/3T3 cells. Briefly, cells were seeded in a 24-well plate and cultured for 12 h, incubated with EXO-DVDMS at a DVMDS concentration of 1 µg/mL for 12 h, then cells were collected and washed thrice with PBS. The cellular DVDMS fluorescence intensity was measured by flow cytometry and quantified by the mean fluorescence intensity of cells.

### Estimation of cell membrane permeability and cellular ROS generation

In order to measure the changes of membrane permeability induced by different treatments, FD500-uptake assay was performed 66. Free-DVDMS or EXO-DVDMS (1 µg/mL DVDMS) were add into the culture medium of 4T1 cells and then sonicated in the presence of 1 mg/mL of FD500. After US (2 W, 60 s), cells were immediately washed with PBS, then the FD500-positive cells were quantified by flow cytometry.

Dichlorofluorescein (DCF) and dihydroethidium (DHE) was used to assess intracellular ROS level and superoxide anion (O_2_•-) production respectively as described in our previous article 50, 67. Briefly, cells were incubated with 10 µM DCHF-DA or 5 µM DHE at 37 °C for 30 min prior to therapeutic treatment. At 2 h after US treatment (3 W, 60 s), the samples were harvested and detected by flow cytometry (Guava easyCyte 8HT, Millipore, Billerica, MA). Multilabel Reader (PE EnSpire, USA) was used to validate the intensity of DHE at an excitation wavelength 520 nm with emission wavelength at 610 nm. For imaging observation, samples were visualized using an E-600 fluorescence microscope.

### Cytotoxicity *in vitro*

For *in vitro* SDT treatment, samples were randomly divided into Control, US, Free-DVDMS, EXO-DVDMS, Free-DVDMS plus US, EXO-DVDMS plus US. Equal DVDMS concentrations in free or exosomal forms at 1, 2, and 4 µg/mL were incubated with 4T1 cells for 12 h, then subjected to US exposure (2W, 3 W, and 5 W, 60 s). The viability was determined at 24 h after different treatment with MTT assay 39. The cell viability was calculated as: Cell survival (%) = OD treatment group/OD control group×100%. Meanwhile, cells after different treatments were fixed with 4% paraformaldehyde solution and stained with calcein-AM/PI.

### *In vivo* blood circulation, biodistribution and homotypic targeting of EXO-DVDMS

In order to detect DVDMS circulation in blood, the BALB/c mice were assigned into two groups and intravenously injected with Free-DVDMS or EXO-DVDMS (2 mg/kg DVDMS). At different time points after injection (5, 10, 15, 30, 45 min, 1, 3, 6, 12, 24 h), the blood samples were collected. The DVDMS content in plasma extraction was measured by fluorescence photometer.

To demonstrate the pharmacokinetics of exosome *in vivo*, DiR was used to label exosome due to its strong red fluorescence in the NIR region. The mice (n = 3) were subcutaneously injected with 4T1 cancer cells (1×10^6^) in flank regions to build xenograft model. After one week, DiR labeled exosomes were injected intravenously and the *in vivo* biodistributions were imaged at 6, 12, 24, 36, 48, 60 and 72 h by IVIS Spectrum Imaging System.

To investigate the homotypic targeting potential of 4T1 cell derived exosomes *in vivo*, we firstly compared EXO-DVDMS accumulation between 4T1 tumor-bearing (homotypic tumor) and CT26 tumor-bearing (non-homotypic tumor) mouse models. The *in vivo* imaging was conducted at 1, 3, 6, 9, 12, 24, 36, 48 and 72 h after 2 mg/kg EXO-DVDMS administration using IVIS Spectrum Imaging System. The organ/tissue-specific distribution of EXO-DVDMS was examined 24 h post injection. To avoid individual differences between mice, a dual-xenograft model with both CT26 tumor and 4T1 tumor were also used in this study. Besides, we prepared blood derived EXO-DVDMS (B-EXO-DVDMS) 68, and compared the tumor accumulation ability of homotypic 4T1 cell derived EXO-DVDMS and non-homotypic B-EXO-DVDMS in 4T1 tumor bearing mouse after 2 mg/kg 4T1-EXO-DVDMS/ B-EXO-DVDMS injection.

### Ultrasound induced tumor targeting, accumulation and permeation of the exosomes

A dual-xenograft model was used to determine the ability of ultrasound to guide exosome accumulation in the tumor tissues. The right-side tumors were sonicated by US1 for 3 min after injection of different particles (DiO labeled exosome, EXO-DVDMS, Free-DVDMS) to increase vascular permeability, while the contralateral tumors were unsonicated. *In vivo* imaging was conducted at 1,3, 6, 9, 12, 24, 36, 48 and 72 h after drug administration. The organ/tissue-specific distribution of the exosomes was examined 24 h post injection, when the tumor-bearing mice were sacrificed and their major organs were excised and imaged.

To determine the efficiency of US triggered drug permeation *in vivo*, the tumor-bearing mice were intravenous injected with DiO labeled exosome (200 µL, 1 mg/mL exosome protein) or EXO-DVDMS (2 mg/kg DVDMS), and only the right tumors were exposed to US1 as described above. The sonicated and the contralateral tumors were collected 24 h later and flash frozen. Continuous cryosections of 10 µm thickness were cut till ~500 µm of the tissue had been sectioned, and the fluorescence distribution of DiO/DVDMS in different depths of the tissues was observed under a Stereo Fluorescence Microscope (Discovery V20, Zeiss, German).

### *In vivo* anti-tumor effects of SDT

To evaluate the anti-tumor efficacy, the 4T1 xenograft mice were divided into ten groups (n = 6): (a) Control, (b) US1+US2, (c) Free-DVDMS, (d) EXO-DVDMS, (e) Free-DVDMS+US1, (f) EXO-DVDMS+US1, (g) Free-DVDMS+US2, (h) EXO-DVDMS+US2, (i) Free-DVDMS+US1+US2, (j) EXO-DVDMS+US1+US2. Free-DVDMS and EXO-DVDMS each with 2 mg/kg DVDMS were injected into the caudal vein, and the mice were exposed to US1 (2 W, 3 min) plus SonoVue® to increase the accumulation of the particles at the target site. 24 h later, the tumors were stimulated with US2 (therapeutic ultrasound at 3 W for 3 min) in order to trigger DVDMS release from the exosomes and initiate a sonodynamic reaction. This procedure was repeated once at the fourth day post the first treatment. The body weight and tumor volume (V= (a × b^2^)/2) were monitored every day. The mice were sacrificed at the end of the experiment, and the tumors were excised, weighed, and processed for various *in situ* histological assays using standard protocols. The tissue sections were suitably stained for PCNA, MMP-9, TUNEL and H&E according to the manufacturers' protocols. In addition, the lungs were removed and fixed in Bouin's solution for 24 h, and the pulmonary nodules were photographed and counted to assess tumor metastasis.

### *In vivo* biocompatibility assessment

The biocompatibility of EXO-DVDMS was evaluated in terms of liver and kidney function. The whole blood and main organs were harvested after the treatment regimen, and assayed for ALT and AST levels (as biochemical makers for acute liver injury), and BUN level (as biochemical maker for acute kidney injury) using commercially available diagnostic kits. The major organs were fixed in formalin, sectioned, and stained with H&E for histopathological analysis.

### Statistical analysis

SPSS 19.0 software (SPSS Inc., Chicago, USA) was used for statistical analysis. All data are expressed as the mean ± standard deviation (S.D.). Differences among the groups were analyzed by one-way ANOVA. A value of p < 0.05 was considered to be of significance.

## Supplementary Material

Supplementary figures and tables.

## Abbreviations

DVDMS: sinoporpyrin sodium
PDT: photodynamic therapy
SDT: sonodynamic therapy
US: ultrasound
RES: reticuloendothelial system
EPR: enhanced permeability and retention
NTA: nanoparticle tracking analysis
PDI: polydispersity index
EE%: encapsulation efficiency
DL%: drug loading
ROS: reactive oxygen species
SOSG: singlet oxygen sensor green
ER: endoplasmic reticulum
AUC: area under the curve
PCNA: proliferating cell nuclear antigen
TUNEL: terminal deoxynucleotidyl transferasemediated dUTP nick-end labeling
MMP: matrix metalloproteinase
CRF%: cumulative released fraction
TA: terephthalic acid
NIR: near infrared spectroscopy
