# Focused ultrasound-augmented targeting delivery of nanosonosensitizers from homogenous exosomes for enhanced sonodynamic cancer therapy: Erratum

**DOI:** 10.7150/thno.78598

**Published:** 2022-11-02

**Authors:** Yichen Liu, Lianmei Bai, Kaili Guo, Yali Jia, Kun Zhang, Quanhong Liu, Pan Wang, Xiaobing Wang

**Affiliations:** Key Laboratory of Medicinal Resources and Natural Pharmaceutical Chemistry, Ministry of Education, National Engineering Laboratory for Resource Developing of Endangered Chinese Crude Drugs in Northwest of China, College of Life Sciences, Shaanxi Normal University, Xi'an, Shaanxi, China.

The authors regret for the inadvertent mistakes in the original version of this article and would like to correct them as below. Free-DVDMS group in Figure 4D and with the presence of US1+US2 in Figure 9F, the biodistributions at 24 h in Figure 7A and 48 h/72 h in Figure S14A, the representative images in Figure S16E were not presented correctly. The authors confirm that the corrections do not affect the descriptions, findings and conclusions reported in the paper. The authors sincerely apologize for any inconvenience caused.

## Figures and Tables

**Figure 4D:**
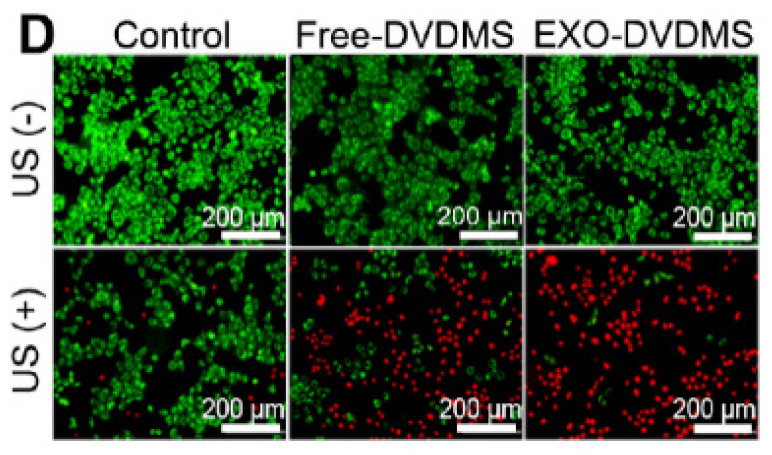
Corrected image.

**Figure 7A:**
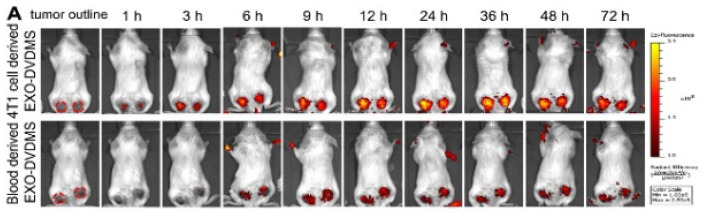
Corrected image.

**Figure 9F:**
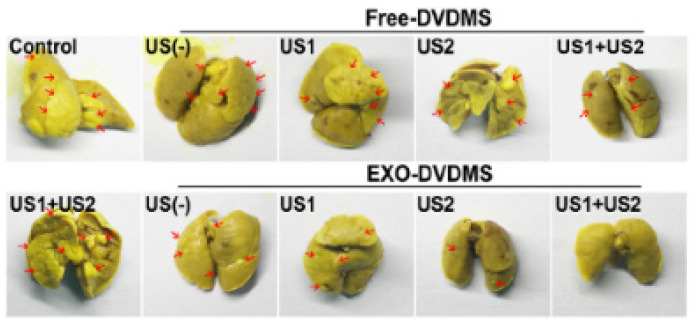
Corrected image

**Figure S14A:**
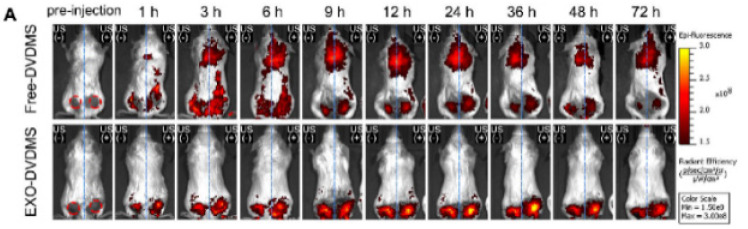
Corrected image.

**Figure S16E:**
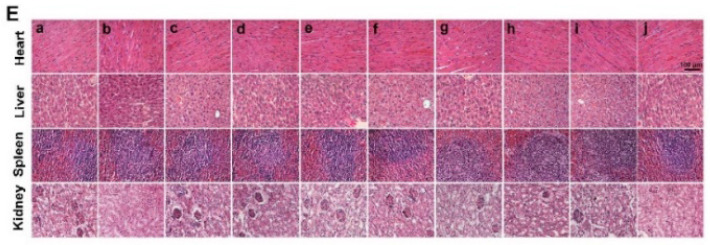
Corrected image.

