# Supplementary material for: Focused ultrasound-augmented targeting delivery of nanosonosensitizers from homogenous exosomes for enhanced sonodynamic cancer therapy
Source: Theranostics. 2019 Jul 9;9(18):5261–81. doi: 10.7150/thno.33183 (PMC6691590; doi:10.7150/thno.33183)
Supplement: Supplementary file 1 — Supplementary figures and tables. [file thnov09p5261s1.pdf]

Supporting Information

Focused Ultrasound-augmented Targeting Delivery of Nanosonosensitizers from Homogenous Exosomes for Enhanced Sonodynamic Cancer Therapy

Yichen Liu†, Lianmei Bai†, Kaili Guo, Yali Jia, Kun Zhang, Quanhong Liu, Pan Wang*, Xiaobing Wang*

All authors are from Key Laboratory of Medicinal Resources and Natural Pharmaceutical Chemistry, Ministry of Education, National Engineering Laboratory for Resource Developing of Endangered Chinese Crude Drugs in Northwest of China, College of Life Sciences, Shaanxi Normal University, Xi'an, Shaanxi, China.

†**Author Contributions:** These authors contributed equally to this work.

***Corresponding Author:**

E-mail: wangxiaobing@snnu.edu.cn

E-mail: wangpan@snnu.edu.cn

Table S1. Mean size, PDI and drug loading capacity of EXO-DVDMS with different mass ratio of DVDMS and exosomes.

Concentration of DVDMS (μg mL^{-1})	DVDMS:EXO (w/w)	EE (%)	DL (%)	DVDMS (μg) /100 ug Protein	Size (nm)	PDI
0	-	-	-	-	112.52 ± 4.68	0.22 ± 0.01
10	1:80.0	74.41	0.90	0.91	115.13 ± 4.21	0.18 ± 0.03
25	1:30.0	70.01	2.27	2.33	118.33 ± 3.11	0.24 ± 0.07
50	1:15.5	84.57	5.18	5.46	126.71 ± 3.86	0.18 ± 0.05
100	1:7.8	61.89	7.40	7.99	211.89 ± 7.94	0.23 ± 0.03
250	1:3.0	31.41	9.45	10.43	432.37 ± 23.57	0.26 ± 0.05

Table S2. Mean size, PDI and drug loading capacity of EXO-DVDMS with different incubation time (0-120 min).

Incubation time (min)	DVDMS:EXO (w/w)	EE (%)	DL (%)	DVDMS (μg) /100 ug Protein	Size (nm)	PDI
0	-	-	-	-	112.52 ± 4.68	0.22 ± 0.01
5	1:15.5	81.78	5.01	5.27	117.24 ± 3.13	0.18 ± 0.04
15	1:15.5	82.51	5.05	5.32	123.65 ± 4.38	0.17 ± 0.03
30	1:15.5	84.57	5.18	5.46	126.71 ± 3.86	0.18 ± 0.05
60	1:15.5	76.45	4.68	4.93	135.75 ± 2.73	0.17 ± 0.04
120	1:15.5	76.93	4.71	4.96	131.32 ± 3.86	0.19 ± 0.04

Table S3. Mean size, PDI and drug loading capacity of EXO-DVDMS at different incubation temperature (4℃, 25℃ and 37℃)

Incubation temperature (℃)	DVDMS:EXO (w/w)	EE (%)	DL (%)	DVDMS (μg) /100 ug Protein	Size (nm)	PDI
**4**	**-**	**-**	**-**	**-**	**112.52 ± 4.68**	**0.22 ± 0.01**
**4**	**1:15.5**	**86.33**	**5.29**	**5.57**	**167.58 ± 8.44**	**0.23 ± 0.05**
**25 (RT)**	**1:15.5**	**84.57**	**5.18**	**5.46**	**126.71 ± 3.86**	**0.18 ± 0.05**
**37**	**1:15.5**	**64.51**	**3.95**	**4.16**	**118.41 ± 4.02**	**0.17 ± 0.03**

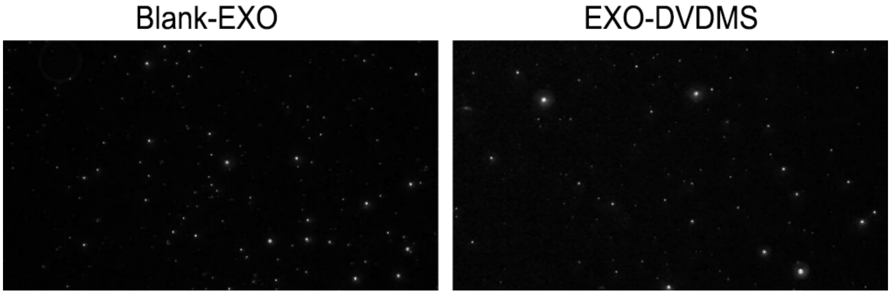

Figure S1. Images captured during NTA measurement.

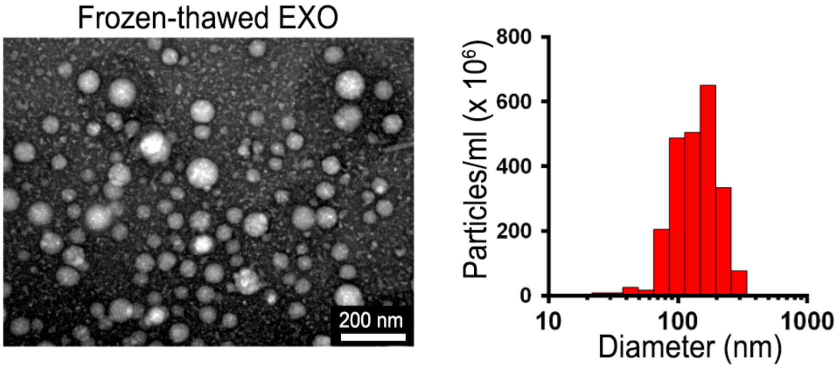

Figure S2. TEM image of Frozen-thawed EXO (left), scale bar = 200 nm. Size distributions histogram of Frozen-thawed EXO based on NTA measurements (right).

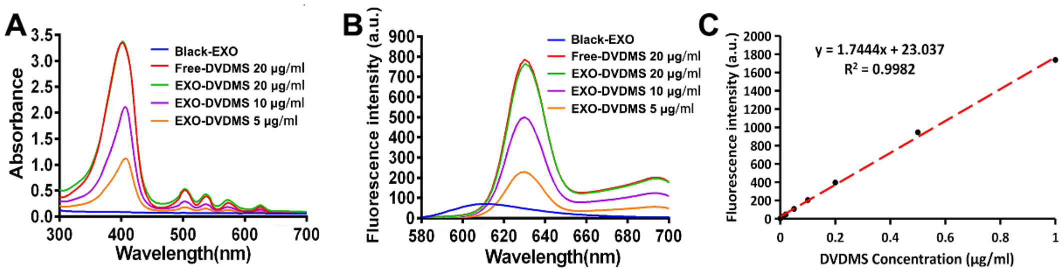

Figure S3. (**A**) Absorption spectra and (**B**) fluorescence spectra of Free-DVDMS, EXO-DVDMS after lysed at DVDMS concentration range from 0-20 μg/mL. (**C**) Standard curve of DVDMS concentration and fluorescence intensity (λex = 410 nm, λem = 627 nm).

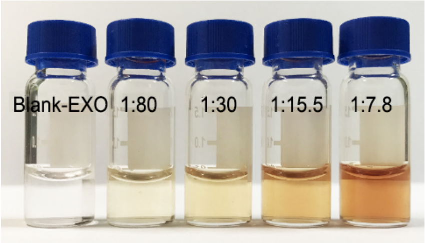

Figure S4. The photographs of Blank-EXO and EXO-DVDMS solutions with different mass radio at the 7th day (4 °C).

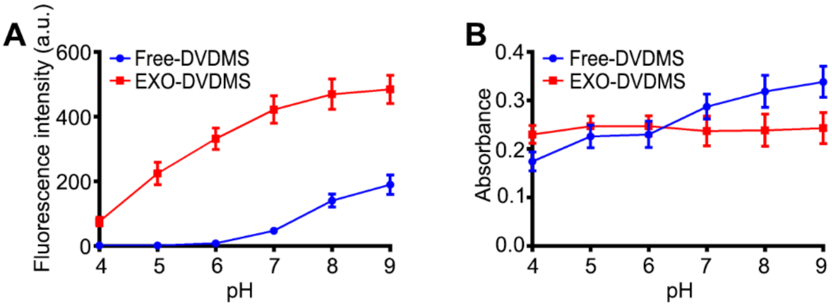

Figure S5. Fluorescence-emission (A) and absorption value (B) of Free-DVDMS and EXO-DVDMS under different storage pH.

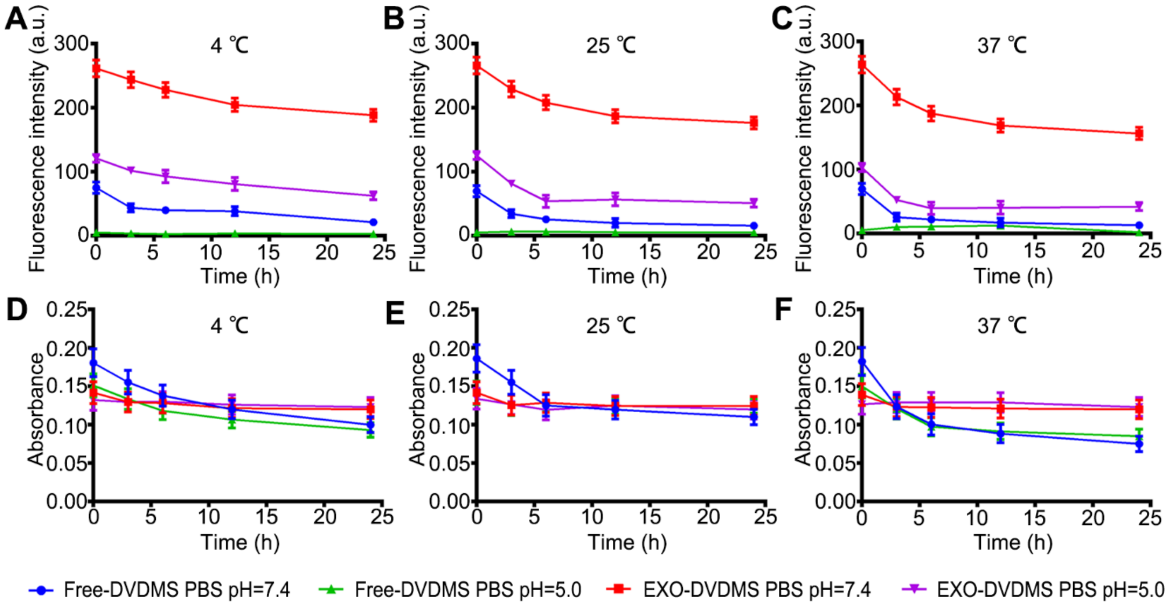

Figure S6. Time course of fluorescence-emission intensity (A, B, C) at 631 nm and absorption value at 410 nm (D, E, F) changes of Free-DVDMS and EXO-DVDMS under different storage temperature (4, 25 and 37℃).

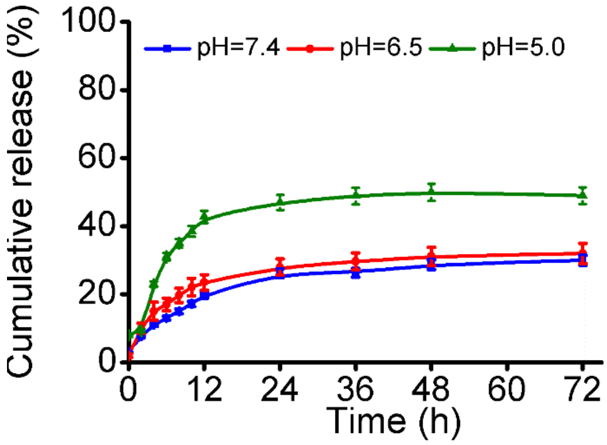

Figure S7. Drug release of EXO-DVDMS under physiological condition (pH 7.4), tumor microenvironment condition (pH 6.5), and late endosomes/lysosomes condition (pH 5.0).

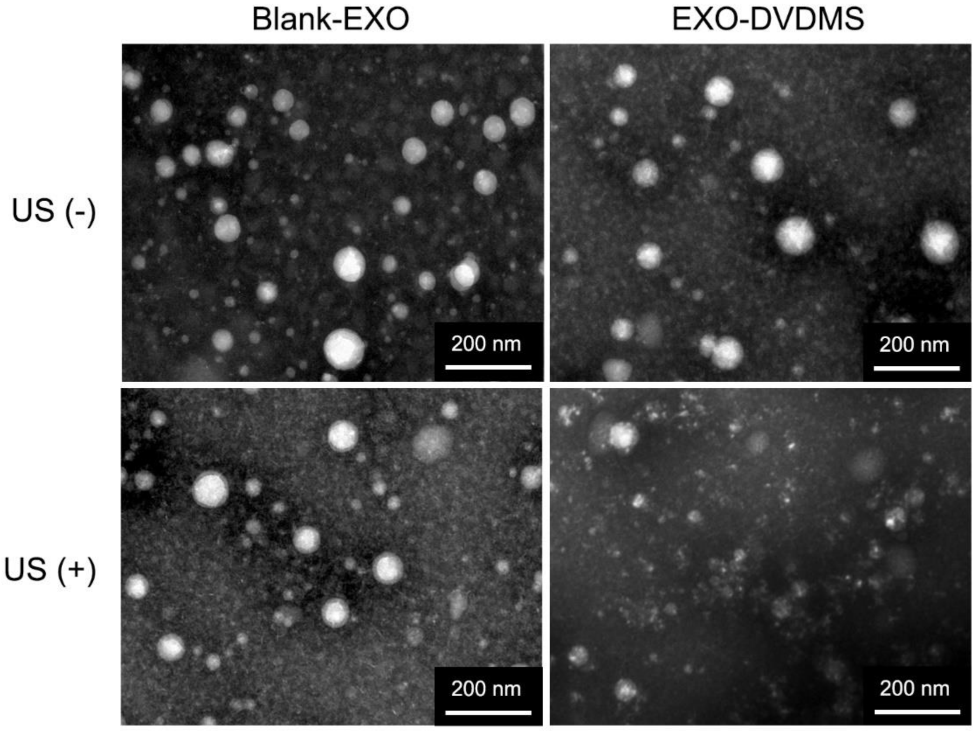

Figure S8 TEM image of Blank-EXO and EXO-DVDMS before and after ultrasound irradiation. Scale bar, 200 nm.

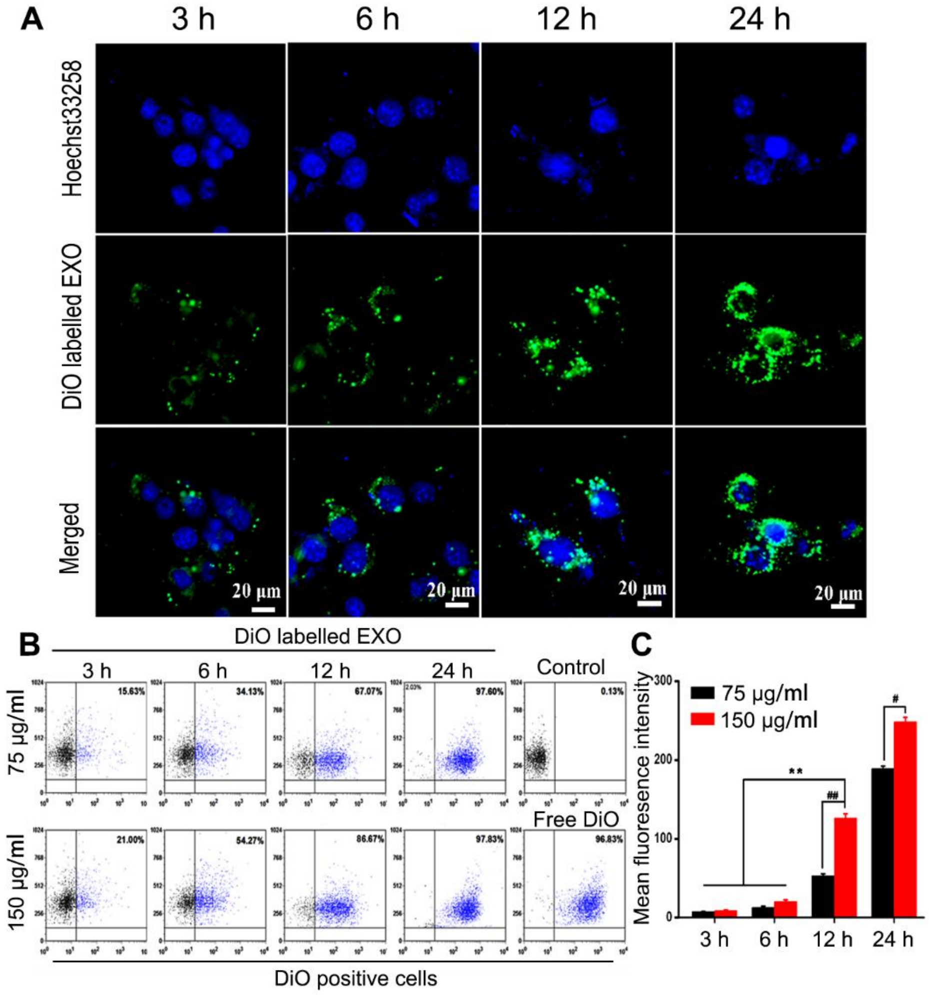

Figure S9. Internalization pattern of DiO labeled exosome by 4T1 cells. (**A**) Intracellular fluorescence was observed using confocal microscopy at the indicated time points (3, 6, 12, 24 h) after incubated with 75 μg/mL exosome. (**B**) Fluorescence of internalized exosome (75 and 150 μg/mL) at 3, 6, 12, 24 h was quantified using flow cytometry analysis. (**C**) Mean fluorescence intensity of flow cytometry results.

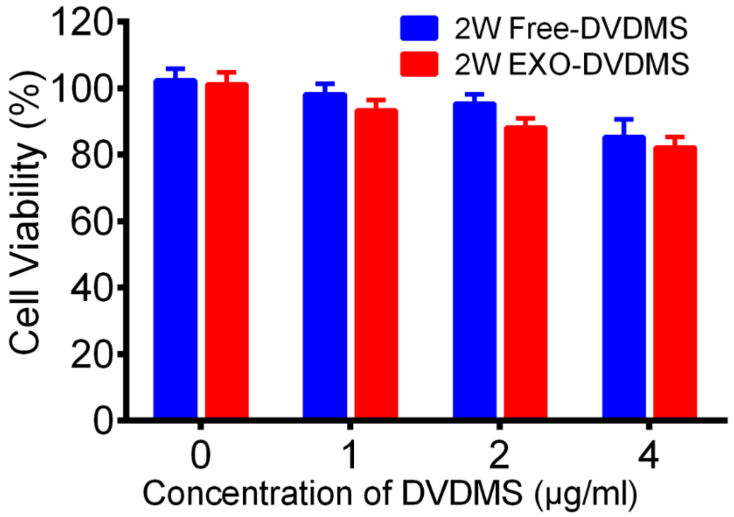

Figure S10. Cell viability on 4T1 cells by Free-DVDMS and EXO-DVDMS after 2W US treatment (irradiation time: 60 s).

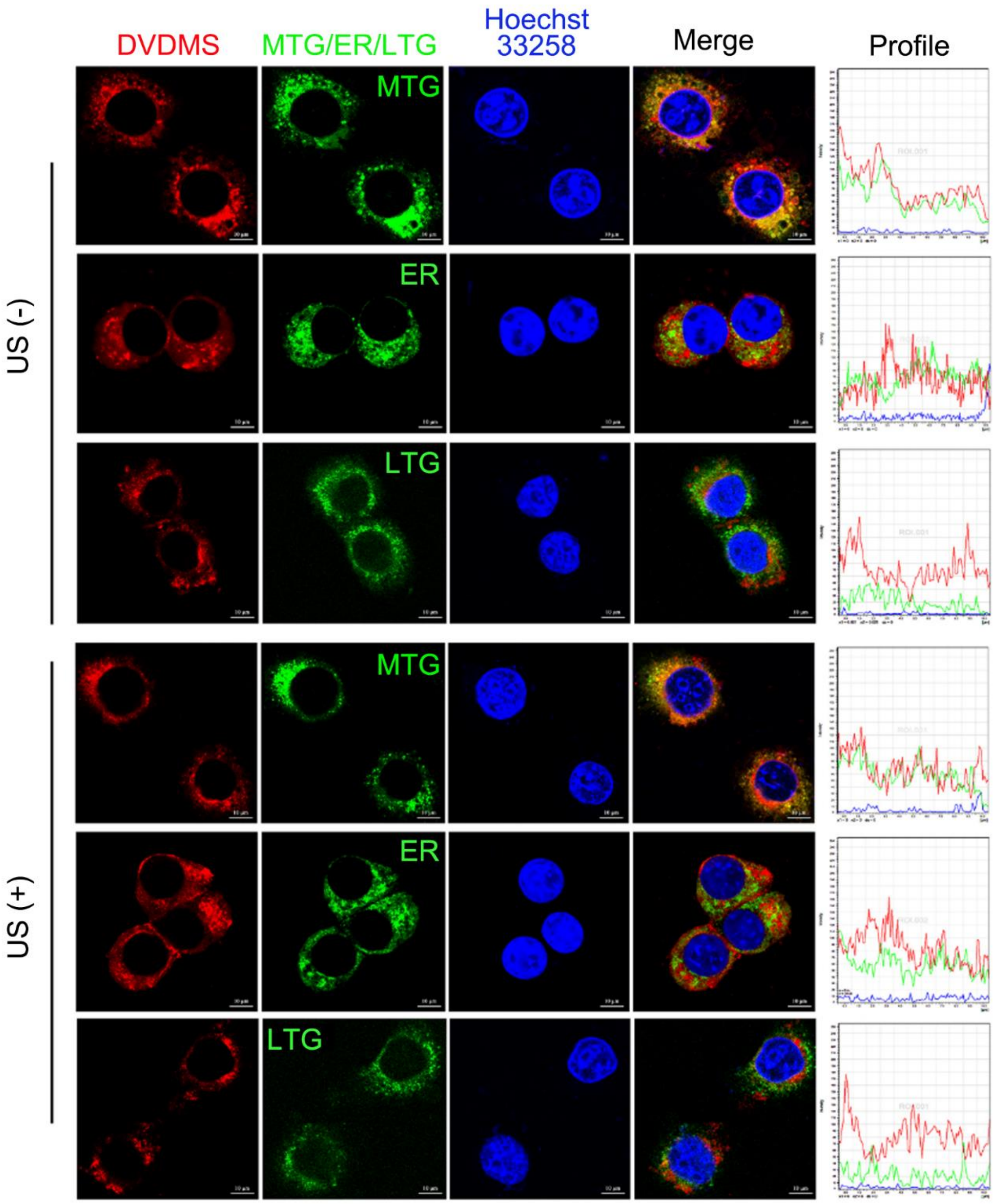

Figure S11. Subcellular localization of Free-DVDMS (serum free) upon ultrasound treatment. 4T1 cells were seeded in chamber slides and incubated for 6 h with 5 μg/mL Free-DVDMS (serum free), then exposed to ultrasound prior to confocal imaging. DVDMS fluorescence (red), MTG, LTG and ER fluorescence (green), nuclear DAPI fluorescence (blue) were captured by confocal microscopy, scale bar=10 μm.

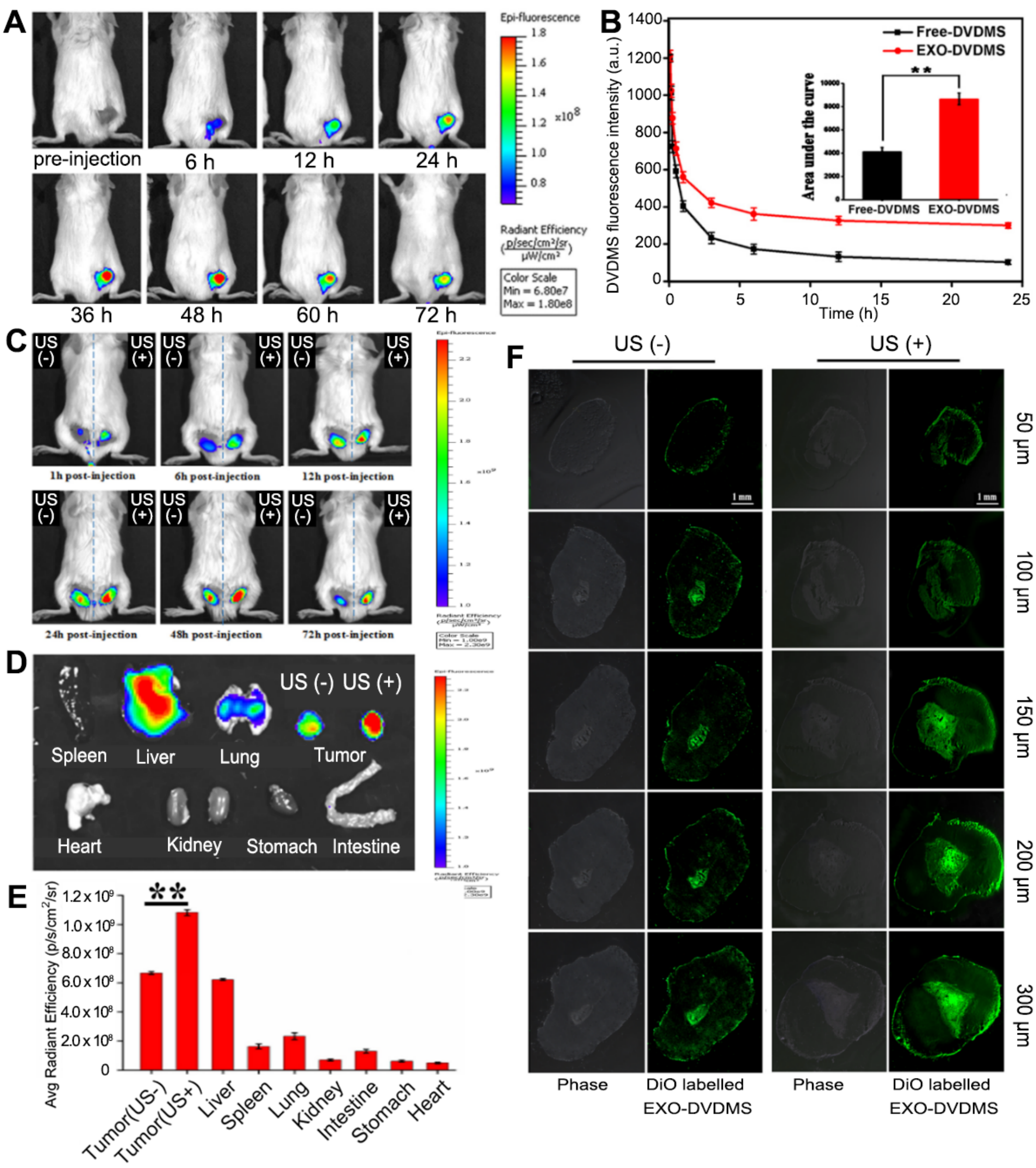

Figure S12. ***In vivo*** **bio-distribution and ultrasound promoted in situ EXO-DVDMS deposition and permeation. (A)** *In vivo* NIR images of subcutaneous 4T1 tumor-bearing mice after intravenous injection of DiR labeled exosomes. **(B)** Blood circulation time of DVDMS after intravenous administration with Free-DVDMS (black) and EXO-DVDMS (red), respectively. $^{**}p<0.01$, between groups. Data shown are mean ± S.D. of three batches. **(C)** *In vivo* NIR images of subcutaneous dual-tumor bearing mice with or without US after intravenous injection of DiR labeled exosomes (200 μg per mouse). Right tumors were exposed to US1 (2 W, 3 min), left tumors without US1 were used for comparison. **(D)** *Ex vivo* NIR images detected biodistribution of tumors and major organs after 24 h of administration. **(E)** Quantitative analysis of fluorescence intensity of tumors and major organs after 24 h of administration (p/s/cm^2/sr). **(F)** US1-assisted drug delivery and penetration after injection with DiO labeled exosomes. $^{**}p<0.01$, between groups. Data shown are mean ± S.D. of three batches.

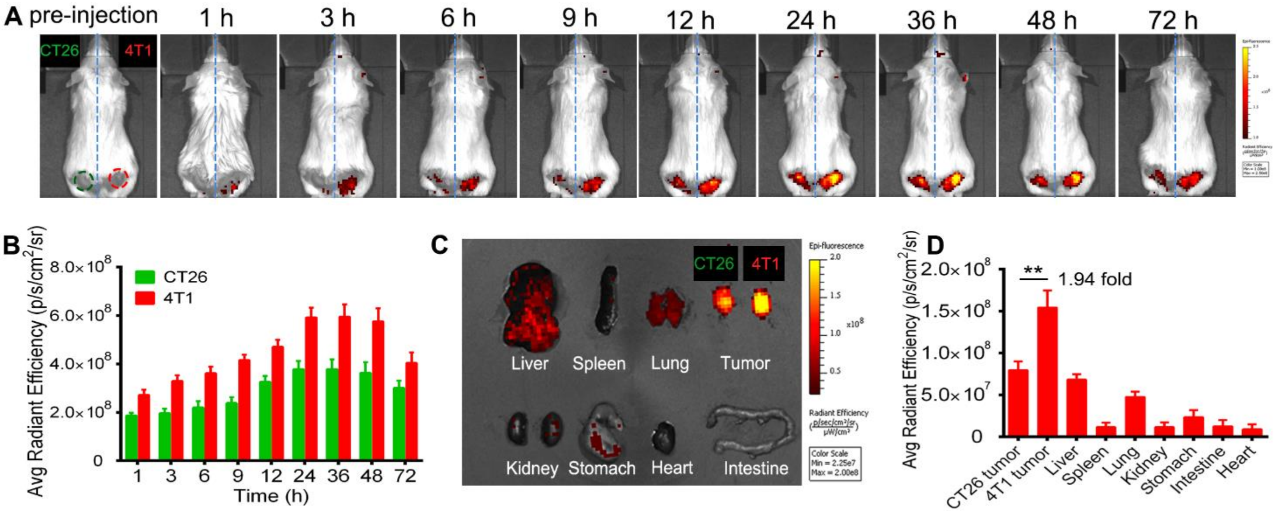

Figure S13. In vivo homotypic targeting potential of EXO-DVDMS to specifically deliver DVDMS to 4T1 tumor tissues using dual-xenograft model. (A) *In vivo* images of subcutaneous 4T1 tumor-bearing (homotypic tumor) and CT26 tumor-bearing (non-homotypic tumor) mice after intravenous injection of and EXO-DVDMS (2 mg/kg). **(B)** Quantification of EXO-DVDMS in 4T1 and CT26 tumors *in vivo.* **(C)** Ex vivo images of tumors and major organs after 24 h of EXO-DVDMS administration. **(D)** Quantitative analysis of fluorescence intensity of tumors and major organs after 24 h of administration (p/s/cm^2/sr). **p<0.01, between groups. Data shown are mean ± S.D. of three batches.

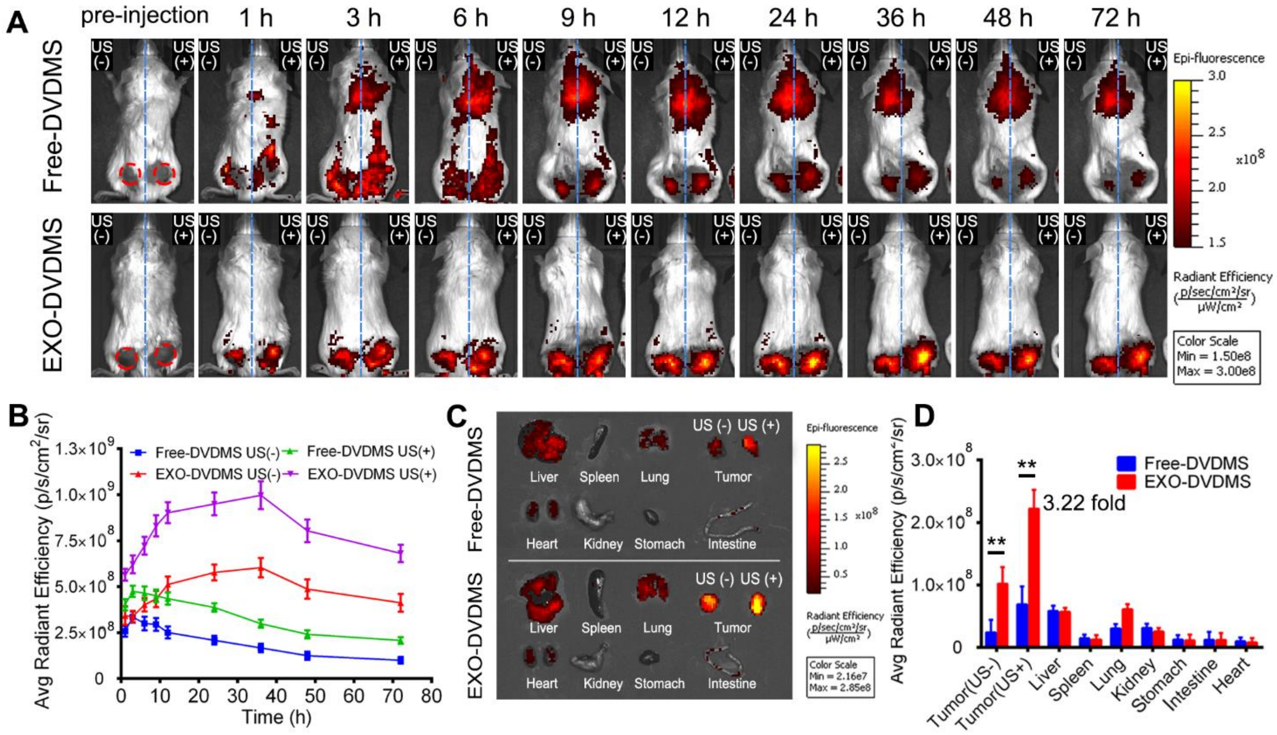

Figure S14. Comparation of *in vivo* bio-distribution of Free-DVDMS and EXO-DVDMS and ultrasound promoted in situ tumor accumulation. (A) *In vivo* images of subcutaneous dual-tumor bearing mice with or without US after intravenous injection of Free-DVDMS and EXO-DVDMS (2 mg/kg). Right tumors were exposed to US1 (2 W, 3 min), left tumors without US1 were used for comparison. **(B)** Quantification of enrichment tendency of EXO-DVDMS in 4T1 tumor *in vivo*. **(C)** *Ex vivo* images detected biodistribution of tumors and major organs after 24 h of Free-DVDMS and EXO-DVDMS administration. **(D)** Quantitative analysis of fluorescence intensity of tumors and major organs after 24 h of administration (p/s/cm^2/sr) **p<0.01, between groups. Data shown are mean ± S.D. of three batches.

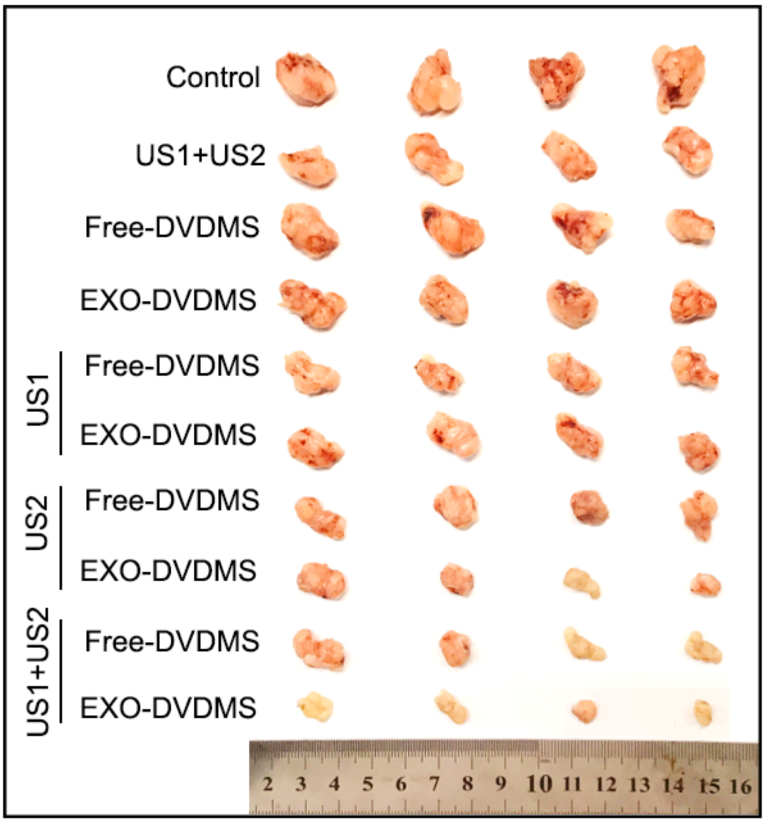

Figure S15. Images of tumors excised from each group of mice 12 days after treatment.

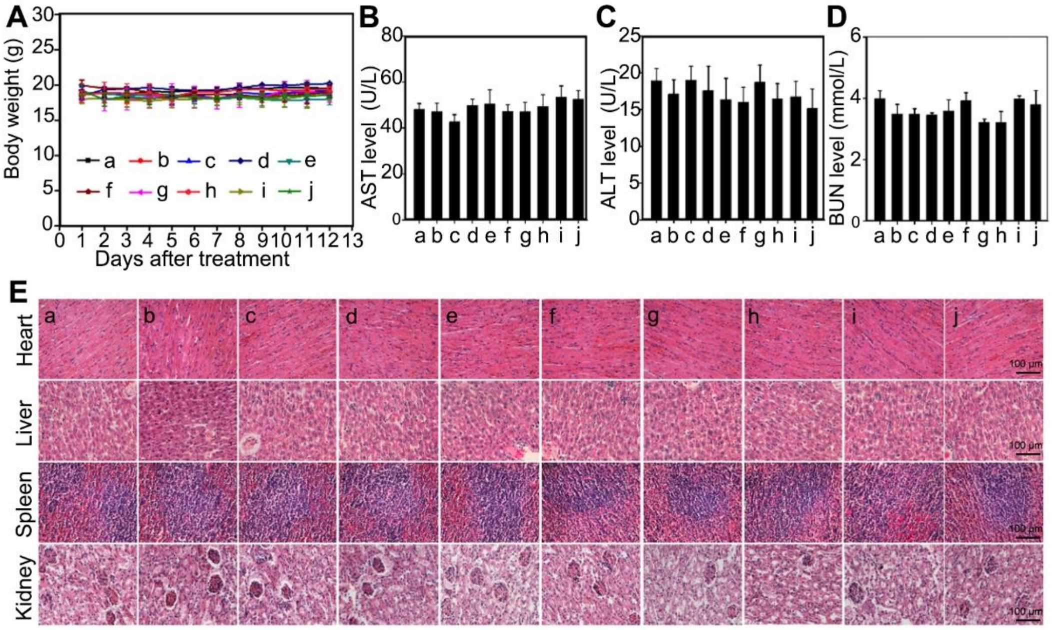

Figure S16. Primary safety evaluation. (**A**) Body weights were measured during the evaluation period in mice under different treatment conditions. (**B**) The AST, (**C**) ALT and (**D**) BUN level in serum of BALB/c mice after different treatment. (**E**) H&E stained major tissues (200×) a: Control, b: US1+US2, c: Free-DVDMS (2 mg/kg), d: EXO-DVDMS (2 mg/kg), e: Free-DVDMS+US1, f: EXO-DVDMS+US1, g: Free-DVDMS+US2, h: EXO-DVDMS+US2, i: Free-DVDMS+US1+US2, j: EXO-DVDMS+US1+US2.
